# Dynamic changes in RNA m^6^A and 5 hmC influence gene expression programs during macrophage differentiation and polarisation

**DOI:** 10.1007/s00018-024-05261-9

**Published:** 2024-05-23

**Authors:** Natalia Pinello, Renhua Song, Quintin Lee, Emilie Calonne, Kun-Long Duan, Emilie Wong, Jessica Tieng, Majid Mehravar, Bowen Rong, Fei Lan, Ben Roediger, Cheng-Jie Ma, Bi-Feng Yuan, John E. J. Rasko, Mark Larance, Dan Ye, François Fuks, Justin J.-L. Wong

**Affiliations:** 1 Faculty of Medicine and Health, The University of Sydney, Camperdown, 2050 Australia; 2 Epigenetics and RNA Biology Program Centenary Institute, The University of Sydney, Camperdown, 2050 Australia; 3 Present Address: Functional Genomics Laboratory, Institut Pasteur de Montevideo, 11400 Montevideo, Uruguay; 4 Laboratory of Cancer Epigenetics, Faculty of Medicine, ULB Cancer Research Center (U-CRC), Jules Bordet Institute, Université Libre de Bruxelles (ULB), Brussels, Belgium; 5 The Molecular and Cell Biology Lab, Key Laboratory of Medical Epigenetics and Metabolism, Institutes of Biomedical Sciences, Shanghai Medical College, Fudan University, Shanghai, China; 6 Shanghai Key Laboratory of Medical Epigenetics, International Co-Laboratory of Medical Epigenetics and Metabolism, Ministry of Science and Technology, Institutes of Biomedical Sciences, and Key Laboratory of Carcinogenesis and Cancer Invasion, Ministry of Education, Liver Cancer Institute, Zhongshan Hospital, Fudan University, Shanghai, 200032 China; 7 Skin Inflammation Group, Centenary Institute, The University of Sydney, Camperdown, 2050 Australia; 8 Present Address: Autoimmunity, Transplantation and Inflammation (ATI) Disease Area, Novartis Institutes for BioMedical Research, Basel, Switzerland; 9 School of Public Health, Wuhan University, Wuhan, 430071 China; 10 Gene and Stem Cell Therapy Program, Centenary Institute, The University of Sydney, Camperdown, 2050 Australia; 11 Cell and Molecular Therapies, Royal Prince Alfred Hospital, Camperdown, 2050 NSW Australia; 12 Charles Perkins Centre, School of Medical Sciences, University of Sydney, Sydney, 2006 Australia

**Keywords:** RNA modifications, Epitranscriptomics, M^6^A, 5 hmC, Macrophages

## Abstract

RNA modifications are essential for the establishment of cellular identity. Although increasing evidence indicates that RNA modifications regulate the innate immune response, their role in monocyte-to-macrophage differentiation and polarisation is unclear. While m^6^A has been widely studied, other RNA modifications, including 5 hmC, remain poorly characterised. We profiled m^6^A and 5 hmC epitranscriptomes, transcriptomes, translatomes and proteomes of monocytes and macrophages at rest and pro- and anti-inflammatory states. Transcriptome-wide mapping of m^6^A and 5 hmC reveals enrichment of m^6^A and/or 5 hmC on specific categories of transcripts essential for macrophage differentiation. Our analyses indicate that m^6^A and 5 hmC modifications are present in transcripts with critical functions in pro- and anti-inflammatory macrophages. Notably, we also discover the co-occurrence of m^6^A and 5 hmC on alternatively-spliced isoforms and/or opposing ends of the untranslated regions (UTR) of mRNAs with key roles in macrophage biology. In specific examples, RNA 5 hmC controls the decay of transcripts independently of m^6^A. This study provides (i) a comprehensive dataset to interrogate the role of RNA modifications in a plastic system (ii) a resource for exploring different layers of gene expression regulation in the context of human monocyte-to-macrophage differentiation and polarisation, (iii) new insights into RNA modifications as central regulators of effector cells in innate immunity.

**Supplementary Information:**

The online version contains supplementary material available at 10.1007/s00018-024-05261-9.

## Introduction

RNA modifications modulate RNA metabolism and influence major biological processes including splicing, RNA nuclear export, RNA decay and translation [1–4]. More than 180 RNA modifications have been identified so far mainly in tRNA and rRNA, and through recognition by effector RNA binding proteins, many of them are pivotal for cellular differentiation, maintenance of cellular identity, and function [5, 6].

N-6 methyladenosine (m^6^A), the most abundant internal RNA modification on messenger RNAs (mRNAs), is widespread across mammalian transcriptomes [5, 7]. m^6^A deposition is catalysed by a methyltransferase (or ‘writer’) complex composed of a catalytic subunit formed by METTL3 and METTL14 [8] and several auxiliary proteins, including VIRMA, WTAP, CBLL1, ZC3H13 and RBM15 [9–12]. Reversal of m^6^A methylation is performed by either of the two known m^6^A ‘eraser’ enzymes, FTO and ALKBH5 [13, 14]. RNA binding proteins known as m^6^A ‘readers’ recognise m^6^A-methylated RNAs to maintain key cellular processes and modulate response to environmental cues [15]. m^6^A is now established as an essential player in physiological processes including development [16], maintenance of cellular identity [17] and immune response [18–20].

5-hydroxymethylcytosine (5 hmC) is one of the least studied RNA modifications. As with DNA, RNA 5 hmC is generated by oxidation of 5mC by the Ten-eleven translocation proteins (TET1, TET2 and TET3) [21–23]. In mammals, RNA 5 hmC has been implicated in infection-induced myelopoiesis [24], degradation of transcripts derived from endogenous retroviruses [25] and stabilisation of pluripotency-promoting transcripts [23]. In contrast to m^6^A, which has been extensively mapped across different tissues, cell types and pathological conditions [26, 27], few studies have explored the roles of RNA 5 hmC in normal physiology and disease states [21, 23]. Recent studies have revealed overlap between the functions of RNA m^6^A and 5 hmC, including the regulation of RNA stability and translation [3, 21, 23, 28]. However, the interplay between m^6^A and 5 hmC in the regulation of biological processes remains unknown.

Investigating multiple RNA modifications simultaneously in the same biological pathway may uncover potential interactions between these modifications. This knowledge will inform whether the absence or presence of a particular RNA modification could impact other modifications and therefore mRNA fate. This understanding will also clarify whether modulating a single RNA modification is sufficient to alter a biological process, or if it necessitates simultaneous modulation of multiple RNA modifications.

Monocytes and macrophages are mononuclear phagocytic cells central to innate immunity [29]. During inflammation, monocytes are recruited to sites of injury or infection where they differentiate into macrophages [30]. Macrophages are remarkably plastic cells with roles in development, homeostasis, tissue repair and immunity [31]. These cells rapidly alter their physiology in response to diverse environmental stimuli and give rise to morphologically and functionally diverse macrophage subpopulations with pro- and anti-inflammatory roles.

The roles of m^6^A methylation during monocyte differentiation into macrophages and subsequent polarisation of macrophages into pro- or anti-inflammatory cells remain elusive. To investigate the role of m^6^A in macrophage polarisation, two recent studies have exposed METTL3-depleted mouse bone marrow-derived macrophages (BMDMs) to pro-inflammatory stimuli. However, one study reported that METTL3 depletion upregulates the expression of pro-inflammatory cytokines, including *TNF* and *IL-6* [32], whereas the other reported a decrease in these cytokines in METTL3-depleted BMDMs [33]. It is conceivable that the levels and duration of METTL3 depletion may influence experimental outcomes, as long-term METTL3 or METTL14 depletion has been reported to induce major transcriptional changes, hence leading to indirect effects [34]. Furthermore, studies using acute and stable depletion of METTL3 in the same embryonic stem cells have yielded opposite results, whereby acute and stable depletion led to silencing and enhancement of retroviral mRNA transcription respectively [35, 36]. Thus, additional and alternative experimental approaches, such as direct profiling of m^6^A during progressive states of macrophage differentiation and polarisation, are necessary to further clarify the involvement of m^6^A in macrophage biology.

TET enzymes that control both DNA and RNA 5 hmC are major regulators of myelopoiesis and immune homeostasis and, when mutated or dysregulated, are linked to the development of haematological malignancies and inflammatory disorders [24, 37–39]. However, the transcriptome-wide distribution of 5 hmC, let alone its function in monocytes and macrophages, has not been explored. The potential roles of RNA 5 hmC and how they affect gene expression together and independently of a prominent RNA modification like m^6^A in macrophage differentiation and polarisation remain to be elucidated.

Due to macrophage heterogeneity and the difficulty in capturing the complexity observed in vivo, generalised in vitro models of pro- and anti-inflammatory macrophages have been developed. A widely used approach is to use the monocytic cell line THP-1. Several studies by us and others have demonstrated that different populations of macrophages derived from this model exhibit similarity in both molecular and immunological signatures to those in primary human monocyte-derived macrophage populations [40–43], providing a suitable system to study RNA modifications in macrophage biology.

**Fig. 1:**
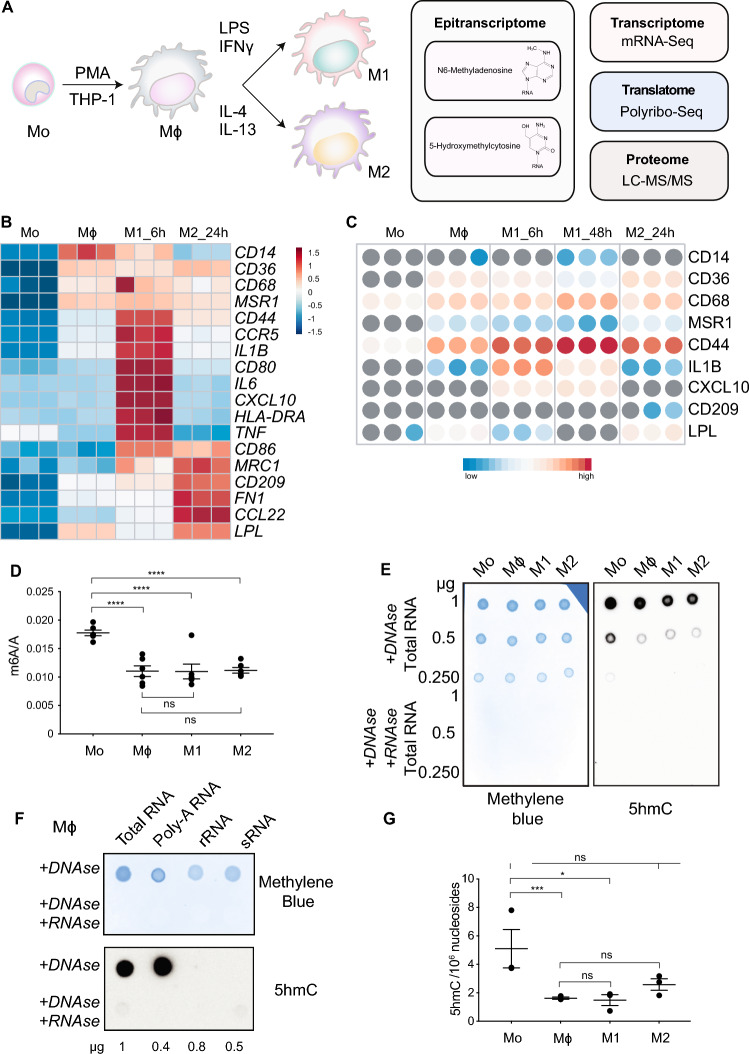
RNA m^6^A and 5 hmC levels decrease during monocyte-to-macrophage differentiation.** A** Diagram detailing our multi-omics approach to study m^6^A and 5 hmC RNA modifications in the THP-1 macrophage differentiation and polarisation model. Heatmaps displaying **B** gene expression (z score) and **C** protein levels of monocytes (Mo), resting-like (Mϕ), pro-inflammatory (M1) and anti-inflammatory macrophages (M2) based on established markers. h indicates hours of polarisation. **D** m^6^A/A on polyadenylated RNAs measured by LC–MS/MS. **E** Dot blot performed with total RNA from Mo, Mϕ, M1 and M2 cells probed with anti-5 hmC antibody (right) and methylene blue loading control (left). Samples were treated with DNase to eliminate the possibility of detecting 5 hmC on DNA. Negative controls were treated with both DNase and RNase A. **F** Dot blot on total RNA, polyadenylated RNA, rRNA and sRNA (including tRNA) from Mϕ cells detected with anti-5 hmC antibody (bottom) or methylene blue loading control (top). Samples were treated with DNase to eliminate the possibility of detecting 5 hmC on DNA. Negative controls were treated with both DNase and RNase A. **G** 5 hmC/10^6 nucleosides in total RNA measured by LC–MS/MS. In **D** and **G**, mean ± SEM is shown. An unpaired two-tailed Student’s t-test was used to determine significance, denoted by ns, not significant; *p < 0.05; ***p < 0.001 and ****p < 0.0001

Here, we obtained transcriptome-wide RNA m^6^A and 5 hmC distributions coupled with gene expression, polyribosome and proteomic profiles from THP-1 monocytic cells and THP-1-derived macrophages at rest and pro- and anti-inflammatory states (Fig. 1A). Through this multi-omics approach, we present data that serves as a reference set for studies on the regulatory roles of RNA m^6^A and 5 hmC in shaping the transcriptomes and proteomes of these cells. We provide evidence that profiling m^6^A changes during progressive stages of macrophage differentiation and polarisation is important to identify key m^6^A-modified genes regulating these processes, which may be otherwise masked if studying changes to gene expression by stably depleting m^6^A-regulatory enzymes alone. We observe the co-occurrence of m^6^A and 5 hmC on mRNAs and speculate that these may regulate the expression, stability and/or alternative splicing of transcripts with crucial roles in macrophage biology. We also provide evidence that 5 hmC can regulate mRNA stability independently of m^6^A. Altogether, our work provides a resource to explore the complex epitranscriptomic processes regulating effector cells of the innate immune system.

## Materials and methods

### Monocyte-to-macrophage differentiation and polarisation

THP-1 cells were maintained in RPMI medium (ThermoFisher Scientific) supplemented with 10% (v/v) fetal calf serum (Hyclone, GE Healthcare), 1% (v/v) non-essential amino acids (ThermoFisher Scientific), 1 mM sodium pyruvate (ThermoFisher Scientific) and 0.1 mg/ml penicillin and streptomycin (ThermoFisher Scientific). To generate THP-1-derived macrophages (Mϕ), THP-1 monocytes (Mo) were stimulated with 100 nM PMA (Sigma) for 48 h. M1 macrophages were generated by stimulating Mϕ with 1 μg/ml LPS (Sigma) and 20 ng/ml IFN-γ for 6 or 48 h. M2 macrophages were generated by stimulating Mϕ with 20 ng/ml IL-4 (R&D Systems) and 20 ng/ml IL-13 (R&D system) for 24 h.

### RNA extraction, cDNA synthesis and RT-qPCR

RNA extraction was performed using TRIzol Reagent (Invitrogen) and cDNA synthesis was performed using iScript gDNA Clear cDNA synthesis kit (Bio-Rad) following the manufacturer’s instructions. RT-qPCR was performed using SensiFAST SYBR No-ROX Kit (Bioline) and 0.3 μM of forward and reverse primers. Samples were amplified and analysed using the LightCycler 480 Instrument II (Roche), cycling conditions: 95 °C for 3 min, followed by 40 cycles at 95 °C for 10 s, annealing at 60 °C for 30 s, and extension at 72 °C for 20 s. Fold change was calculated using the ΔΔCT method. See Table S1 for a list of the primers used in this work.

### m^6^A and 5 hmC quantification

Sample preparation for m^6^A quantification was performed as described previously [44]. In brief, mRNA was isolated using the Magnetic mRNA Isolation Kit (New England Biolabs), followed by rRNA depletion using the RiboMinus Eukaryote System v2 (Invitrogen) as per manufacturer’s instructions. 200 ng mRNA were digested with 1U nuclease P1 (Sigma) in 20 mL buffer containing 25 mM NaCl, 2.5 mM ZnCl_2_ at 37 °C for 2 h, followed by the addition of 1 M NH_4_HCO_3_ (2 mL) and 1 U of alkaline phosphatase (Sigma). The solution was incubated at 37 °C for 2 h, centrifugated at 13,000 g for 10 min at 37 °C, and then 10 μL of the solution were injected into the mass spectrometer. Quantification was performed by comparison with a standard curve obtained from pure nucleoside standards. The m^6^A to A ratio was calculated based on obtained concentrations.

To quantify global RNA 5 hmC levels, LC–MS/MS was performed as previously described [23]. Briefly, 10X buffer (500 mM Tris–HCl, 100 mM NaCl, 10 mM MgCl_2_, 10 mM ZnSO_4_, pH 7.0), 180 U of S1 nuclease, 0.001 U of venom phosphodiesterase I, and 30 U of CAIP were added to 10 μg of total RNA. The mixture was incubated at 37 °C for 4 h. The resulting solution was then extracted with chloroform three times. The upper aqueous phase was collected and passed through a solid-phase extraction cartridge filled with 50 mg of sorbent of graphitised carbon black to remove the salts. The elution was then dried with nitrogen gas at 37 °C for subsequent chemical labelling and LC–ESI–MS/MS analysis by an AB 3200 QTRAP mass spectrometer (Applied Biosystems).

### mRNA sequencing

Paired-end mRNA-Seq was performed for all samples. 2 µg of DNAse treated total RNA was sent to Novogene for directional mRNA library preparation (mRNA enrichment) and sequencing using NovaSeq. 50 million paired-end 150 bp reads were obtained for each sample.

### Polyribo-Seq

Polyribo-Seq sample preparation and uHPLC Size Exclusion Chromatography (SEC) were performed as described by Yoshikawa et al. [45]. Briefly, cell lysates in CHAPS buffer containing RNAse inhibitors were separated by size exclusion chromatography on a Thermo Dionex BioRs UHPLC and Agilent SEC-5 7.8 × 300 mm HPLC column with 2000 Å pores and 5 mm particles. Polysome fractions were harvested in TRIzol LS reagent (Invitrogen) and RNA was extracted following the manufacturer’s protocol. RNA-Seq was then performed on RNA from the polysome-bound fraction.

### Proteomic analysis

Sample preparation, LC–MS/MS and analysis of spectra was performed as we previously described [42]. Briefly, 20 μg of protein was denatured, reduced and alkylated followed by trypsin digestion at 37 °C for 16 h. Peptides were then purified using StageTips. Dried peptides were resuspended in 60 μL of 5% formic acid and stored at 4 °C until analysed by LC–MS. Using a Thermo Fisher RSLCnano, peptides were separated using a 50 cm × 75 µm C18 (Dr Maisch, Ammerbuch, Germany, 1.9 μm) column with a ∼10 μm pulled tip, coupled online to a nanospray ESI source. Peptides were resolved over a gradient from 5% acetonitrile to 40% acetonitrile over 140 min with a flow rate of 300 nL min ^−1^. Tandem mass spectrometry analysis was carried out on a Fusion Lumos mass spectrometer (Thermo Fisher) using HCD fragmentation. The data-dependent acquisition method used acquired MS/MS spectra of the top 20 most abundant ions at any one point during the gradient. Raw data were analysed using MaxQuant (38) (version 1.6.3.4). Peptide and protein level identification were set to a false discovery rate (FDR) of 1% using the human Uniprot database. Mass tolerance was set to 4.5 ppm for precursor ions and MS/MS mass tolerance was 20 ppm. Enzyme specificity was set to trypsin, with a maximum of 2 missed cleavages permitted. Deamidation of Asn and Gln, oxidation of Met, pyro-Glu and protein N-terminal acetylation were set as variable modifications. Carbamidomethyl on Cys was searched as a fixed modification.

### m^6^A-IP-Seq

m^6^A-IP-Seq was performed as previously described [46]. Briefly, 5 µg of DNAse treated total RNA were subjected to fragmentation with ZnCl_2_ incubation at 70 °C for 13 min. Following precipitation, fragmented RNA size distribution was assessed using RNA 6000 Nano Bioanalyzer kit (Agilent). Approximately 500 ng of sample were stored as input control. Fragmented RNA was subjected to two rounds of m^6^A immunoprecipitation for 2 h each using an anti-m^6^A antibody (ABE572, Merck) previously conjugated to protein-A magnetic beads (Thermo Fisher Scientific) and protein-G magnetic beads (Thermo Fisher Scientific) by incubation at 4 °C for at least 6 h. Following extensive washing, RNA was eluted from the beads using RLT buffer and RNeasy mini kit (Qiagen). RNA was quantified using RNA 6000 Pico Bioanalyzer kit (Agilent). To confirm m^6^A enrichment, cDNA was synthesised using the SensiFast cDNA synthesis kit (Bioline) and SETD7 and GAPDH levels measured by qPCR were used as positive and negative control respectively. Finally, library preparation was performed using the SMARTER Stranded Total RNA Seq kit v2-Pico Input Mammalian kit (Takara Bio) following the manufacturer’s instructions. Libraries were then sequenced using HiSeq2500 (Illumina) and a minimum of 20 million paired-end reads were obtained per sample. This experiment was performed in duplicates for each sample.

### 5hmC-IP-Seq

5 hmC-IP-Seq was performed as described by Delatte, et al. [21] and Lan et al. [23]. In brief, 1 mg of DNAse treated total RNA was chemically fragmented by incubating RNA in fragmentation buffer (10 mM Tris–HCl pH7, 100 mM ZnCl_2_) at 94 °C for 40 s, the reaction was stopped by the addition of 50 mM EDTA. Fragmented RNA was ethanol precipitated and resuspended in nuclease-free water. Fragmentation efficiency was checked by running a Nano RNA Bioanalyzer chip (Agilent) on the Agilent 2100 Bioanalyzer. Prior to immunoprecipitation, fragmented RNA was denatured by incubation at 70 °C for 5 min and then placed on ice. RNA was then incubated at 4 °C overnight with or without 12.5 μg of anti-5 hmC antibody (C15220001, Diagenode) in freshly prepared 1X Immunoprecipitation (IP) buffer (50 mM Tris–HCl pH 7.4, 750 mM NaCl and 0.5% Igepal CA-630, RNasin 400 U/ml and RVC 2 mM) supplemented with protease inhibitor (complete EDTA free, Roche). 60 μL of equilibrated Dynabeads Protein G (Invitrogen) were added to each sample and incubated for 2.5 h at 4 °C. After three washes with 1X IP buffer, samples were eluted by addition of 1 mL of TriPure Reagent (Roche) as per manufacturer’s instructions. Samples were analysed by deep sequencing. Libraries were prepared using the TruSeq ChIP Sample Prep Kit (Illumina) after reverse transcription of pulled-down RNA and synthesis of a second strand (NEBNext mRNA second strand synthesis module (NEB). Briefly, 5 to 10 ng of dsDNA underwent 5′ and 3′ protruding end repair. Then, non-templated adenines were added to the 3′ ends of the blunted DNA fragments. This last step allows ligation of Illumina multiplex adapters. The DNA fragments were size selected to remove unligated adapters and to sequence fragments of 200–300 bp of length. The library was amplified through 18 cycles of PCR. DNA was quantified using Qubit, and DNA integrity was assessed by running a DNA Bioanalyzer chip (Agilent) on the Agilent 2100 Bioanalyzer. 1.5 pM of DNA library spiked with 1% PhiX viral DNA was clustered and sequenced on a NextSeq500 (Illumina). hmeRIP-Seq experiments were performed in triplicates for each condition.

### Enrichment of polyadenylated RNA, rRNA and sRNA

Polyadenylated RNA was enriched using the Poly(A)Purist MAG Kit (Invitrogen) as per manufacturer’s instructions. rRNA was separated from polyA RNA using GeneElute mRNA Miniprep kit (Sigma) and concentrated using RNA Clean and Concentrator kit (Zymo Research) and sRNA (including tRNA) was extracted using miRNeasy mini-Kit (Qiagen) and RNeasy MiniElute Cleanup Kit (Qiagen). RNA quality and purity of the fractions was performed using Small RNA, PICO and NANO RNA Bioanalyzer kits (Agilent).

### 5hmC dot blotting

To perform dot blot, RNA was treated with TURBO DNase (ThermoFisher) following the manufacturer’s protocol. As a control, RNA was treated with 1U RNase A (Qiagen) for 1 h at 56 °C. RNA was then loaded onto a Hybond-N + membrane (GE Healthcare), allowed to air dry and crosslinked twice at 200,000 μJ/cm^2^ UV. To control for RNA loading, the membrane was then incubated with 0.04% methylene blue in 0.5 M sodium acetate for 5 min, following rinsing with PBS 0.1% Tween-20, it was imaged using the colorimetric function of ChemiDoc Imaging System (BioRad). The membrane was then blocked in 3% w/v skim milk PBS-0.1% Tween-20 for 1 h at room temperature, incubated in blocking buffer with 1:500 rat anti-5 hmC (Diagenode) overnight at 4 °C and followed by incubation with 1:1000 anti-rat IgG HRP (ab6734, Abcam) in blocking buffer. 5 hmC detection was performed using SuperSignal West Femto Maximum Sensitivity Substrate (ThermoFisher) and imaged on a ChemiDoc Imaging System (BioRad).

### Flow cytometry

Flow cytometry analysis was performed using the following cell surface markers: anti-CD11b conjugated to phycoerythrin (PE, BD Biosciences, clone ICRF44) and anti-CD44 allophycocyanin (APC, BD Biosciences, clone IM7) conjugated to staining to confirm the differentiation of THP-1 Mo into Mϕ. Anti-CD38 conjugated to PE-Cy7 (BioLegend, clone HIT2) and anti-CD80 conjugated to V450 (BD Biosciences, L307.4) to confirm the polarisation into M1-like cells. Anti-CD209 conjugated to BV421 (BD Biosciences, DCN46) to confirm the polarisation into M2-like cells. Cell viability was determined by staining cells with 0.5 μg/ml DAPI (Invitrogen). Acquisition was performed using a BD FACSCanto™ II and a BD FACSFortessa™ II (BD Biosciences) and data were analysed using FlowJo software (BD Biosciences).

### Generation of METTL3 knock-down cell lines

Stable gene knockdown of METTL3 in THP-1 cells was achieved by lentiviral transduction of the pLKO.1 containing specific short hairpin RNAs (Table S1). The transduced cells were subjected to selection by 7 days culture in 0.6 μg/mL puromycin together with control cells transfected with pLKO.1 transduced with a non-targeting shRNA against an *Arabidopsis Thaliana* gene. Knockdown was confirmed by western blotting.

### RNA decay assay

METTL3-depleted and control THP-1-derived macrophages were treated with 10 µg/ml actinomycin D (Sigma) for 2, 4 and 8 h and harvested. For RNA 5 hmC depletion assays, THP-1-derived macrophages were treated with DMSO or 3 mM Itaconic Acid (Sigma) for 10 h prior to actinomycin D treatment. RNA was extracted using TRIzol. cDNA and qPCR were performed as described above. mRNA decay rate was estimated by non-linear regression curve fitting as previously described [47].

### Bioinformatic analysis

For m^6^A- and 5 hmC-IP-Seq, raw reads were trimmed to remove adaptor sequences and low-quality reads using Trimmomatic with the default setting [48]. Read quality was assessed using FastQC. Clean reads were aligned to the human reference genome hg38 (ENSEMBL version 86) using the STAR aligner [49]. To minimise the rate of false positives, only uniquely mapped reads were selected using samtools [50]. Peaks enriched in immunoprecipitated over corresponding input samples were called using MACS2 [51]. Peaks identified in both biological replicates were merged using the mergePeaks command in the HOMER software [52] and overlapping peaks were mapped to the RefSeq gene annotation using intersectBed from BEDTools [53]. Enriched m^6^A and 5 hmC motifs were identified using de novo motif search with the HOMER software (version 4.9.1). Motifs with the most significant *P*-values were visualised using WebLogo [54]. The metagene profiles were plotted using the ‘Guitar’ R package [55]. DESeq2 [56] was used to identify m^6^A or 5 hmC peak levels that were significantly different (1.5-fold) between two samples (i.e.Mo/Mϕ, Mϕ/M1 and Mϕ/M2), with a Benjamini–Hochberg correction (*p* < 0.05), following normalisation against input control using the design =  ~ group + condition + group:condition function (i.e. (Mϕ_IP/ Mϕ_Input)/(Mo_IP/Mo_Input). The levels of fold-change in m^6^A peaks were further normalised against fold-change in genes expression, enrichment of transcripts in polysomes and protein level changes between samples to identify their associations with m^6^A changes. Gene ontology (GO) biological processes (BP) enrichment analysis was performed for the genes with significantly increased/decreased m^6^A peaks using the ‘clusterProfiler’ package [57].

For mRNA- and Polyribo-Seq, Truseq3-PE adapter and poor-quality sequences were assessed using FastQC were trimmed by Trimmomatic using the default settings [48]. Trimmed reads were then aligned to the hg38 (ENSEMBL version 86) reference genome using STAR. FeatureCounts [58] was subsequently employed to convert aligned short reads into read counts for each sample. The data were then analysed using R and DESeq2 [56]. Differentially enriched mRNAs undergoing translation in the polysomes were identified using Wald statistical test, with fold-change > 1.5 and *p* < 0.05 after Benjamini–Hochberg correction. Expressed genes were identified as those with RPKM greater than 1 for at least one group of samples. Differentially expressed genes between two groups were identified using Wald statistics, with fold-change > 1.5 and *p* < 0.05 after Benjamini–Hochberg correction. The quality and reproducibility of experimental replicates are detailed in our previous publication [59].

### Statistical analysis

Unless stated otherwise, statistical analyses were performed using GraphPad Prism 9.0. Statistical significance was determined using Student’s t-test unless indicated otherwise in figure legends and text. *p* < 0.05 was considered statistically significant. All error bars represent the standard error of the mean (SEM) from independent experiments (n >  = 3 unless specified otherwise).

## Results

### Transcriptomic and proteomic profiling reveal a robust macrophage differentiation and polarisation model derived from THP-1 cells

To study m^6^A and 5 hmC RNA modifications during monocyte-to-macrophage differentiation and polarisation, we differentiated and polarised THP-1 monocytic cells into four cellular stages generated as follows: Untreated THP-1 cells (Mo); Phorbol 12-myristate 13-acetate (PMA)-differentiated cells (Mϕ); PMA-differentiated cells plus lipopolysaccharide (LPS) and interferon-γ (IFNγ) (M1); and PMA-differentiated cells plus IL-4 and IL-13 (M2) (Fig. 1A).

In line with previous reports [42], we observed upregulation of cell surface markers and expression of genes characteristic of differentiated (Fig. S1A and S1B) and polarised (Fig. S1C and S1D) macrophages.

We performed deep mRNA-Seq and liquid chromatography with tandem mass spectrometry (LC–MS/MS) in Mo, Mϕ, M1 and M2. Differences in global gene and protein expression patterns were evident by Principal Component Analysis (PCA) of mRNA-Seq (Fig. S1E) and LC–MS/MS (Fig. S1F) data. For M1, LC–MS/MS was performed at 6 and 48 h post-stimulation to capture dynamic proteomic changes associated with macrophage reprogramming in response to LPS and IFNγ stimulation [60]. This analysis showed a clear separation of monocytes from macrophages and highlighted M1 as a highly distinct subset within our modelled macrophages. As expected, we observed clustering of technical replicates within each cellular stage. We further confirmed the identities of the four cell types via gene expression analysis (Fig. 1B) and proteomics profiling (Fig. 1C) of established cell type-specific markers [41, 42, 58, 61–64]. Overall, our data indicate that the THP-1 macrophage differentiation and polarisation model is robust in our hands. Importantly, these transcriptomic and proteomic profiles resemble, at least in part, those of human primary monocytes and macrophages [65]. Therefore, we concluded this system was suitable to study RNA modifications in monocytes and macrophages*.*

### Global levels of RNA m^6^A and 5 hmC decrease during macrophage differentiation

To investigate changes in global m^6^A levels during monocyte-to-macrophage differentiation and polarisation, we quantified m^6^A on polyadenylated mRNAs from Mo, Mϕ, M1 and M2 cells by LC–MS/MS. We found that m^6^A levels on mRNA decreased during the transition of monocytes to resting macrophages and remained unchanged when macrophages were treated with polarising stimuli (Fig. 1D).

To investigate global RNA 5 hmC changes during macrophage differentiation and polarisation, we first performed dot blots. We found that total RNA 5 hmC levels decreased in macrophages compared to monocytes (Fig. 1E). This decrease is likely due to changes in 5 hmC levels on mRNAs, as we observed higher 5 hmC signal on poly-A-RNA compared to total RNA while we did not detect 5 hmC signal in ribosomal (rRNA) or small (sRNA) fractions (Fig. 1F). No 5 hmC signal was detected on rRNA or sRNA by dot blot (Fig. 1F). Higher global 5 hmC levels in total RNA of monocytes compared to macrophages was further confirmed using LC–MS/MS (Fig. 1G). Like m^6^A, global 5 hmC levels significantly decreased during monocyte-to-macrophage differentiation, and no significant difference was observed during polarisation. In summary, global m^6^A and 5 hmC RNA levels decrease during monocyte-to-macrophage differentiation and remain stable following exposure to pro- and anti-inflammatory stimuli.

### Transcriptome-wide profiling of m^6^A reveals its association with transcription and translation of genes involved in monocyte-to-macrophage differentiation and polarisation

**Fig. 2:**
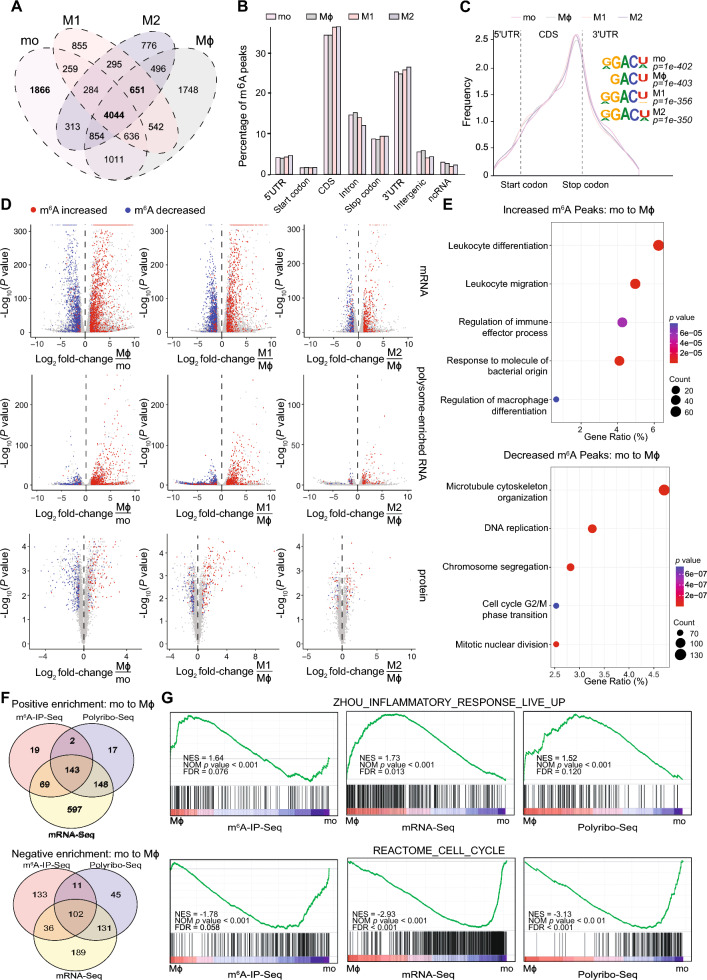
m^6^A is associated with gene functions involved in monocyte-to-macrophage differentiation and polarisation. **A** Venn diagram showing the overlap of m^6^A peaks found in THP-1 monocytes (Mo), resting-like (Mϕ), pro-inflammatory (M1) and anti-inflammatory macrophages (M2). **B** Percentage of m^6^A peaks identified by m^6^A-IP-Seq in defined transcript and genomic regions: coding sequence (CDS), 5′ untranslated region (5′UTR), 3′ untranslated region (3′UTR), start and stop codons, intergenic regions and non-coding RNA (ncRNA) in Mo, Mϕ, M1 and M2. **C** Metagene profiles detailing m^6^A distribution along a normalised transcript and top enriched sequence motifs identified by m^6^A-IP-Seq in all cell types. **D** Volcano plots showing differential gene expression (mRNA, top), translation (polysome-enriched RNA, middle) and protein levels (bottom). Red and blue dots represent individual genes with increased and decreased m^6^A peaks respectively in Mϕ compared to Mo, M1 compared to Mϕ and M2 compared to Mϕ. **E** Gene ontology analysis of increased (top) and decreased (bottom) m^6^A peaks during Mo to Mϕ differentiation. **F** Venn diagram showing overlapping gene sets identified by Gene Set Enrichment Analysis (GSEA) on m^6^A-Seq, mRNA-Seq and polyribo-Seq data presenting positive (top) or negative (bottom) enrichment during Mo to Mϕ differentiation. **G** GSEA signatures showing significantly positive (top) or negative (bottom) enrichment during Mo to Mϕ differentiation

To determine transcripts that are modified by m^6^A in monocytes and macrophages, we performed m^6^A RNA immunoprecipitation sequencing (m^6^A-IP-Seq) in Mo, Mϕ, M1 and M2 cells. We identified 8511–12,425 m^6^A peaks (corresponding to 6118–7130 genes) in monocytes and macrophages (Table S2), of which 4044 m^6^A peaks (corresponding to 4185 genes) were common to all four cellular stages. 1866 m^6^A peaks (in 747 genes) were exclusive to monocytes and 651 m^6^A peaks (in 507 genes) were present in all types of macrophages (Fig. 2A). Overall m^6^A profiles obtained were consistent with previous studies [7, 66], whereby half (~ 45%) of the m^6^A peaks identified in both monocytes and macrophages were present in the coding sequence (CDS) while approximately 30% were located in the 3′ untranslated region (3′ UTR) (Fig. 2B). Metagene profiles of all four cellular stages presented the characteristic m^6^A distribution along the mRNA transcript with a marked enrichment of this modification preceding the stop codon (Fig. 2C). As expected, sequence logo analysis revealed the canonical DRACH motif in all four data sets (Fig. 2C).

By normalizing m^6^A peak levels to gene expression changes, we found that m^6^A methylation during macrophage differentiation and polarisation was enriched in specific transcripts with increased expression and vice versa (Fig. 2D). Similar enrichment was observed between transcriptome-wide m^6^A levels and protein translation as measured by polyribosome sequencing (Polyribo-Seq) and LC–MS/MS (Fig. 2D). These results support previous reports showing that m^6^A is coupled to transcription and/or promotes translational processes [67–70].

To identify a functional association between differential m^6^A modification and macrophage differentiation and/or polarisation, we performed Gene Ontology (GO) analysis on differential m^6^A peaks. While most mRNAs presenting differential m^6^A methylation (n = 6141) showed decreased m^6^A peaks in Mϕ compared to Mo, ~ 30% of transcripts showed increased m^6^A peaks (n = 1904) in Mϕ. mRNAs with increased m^6^A peaks in Mϕ compared to Mo showed enrichment for functions associated with macrophage differentiation and immune response (Fig. 2E). Consistent with the cessation of cell proliferation typical of differentiated Mϕ [71], transcripts related to cell cycle, DNA replication and chromosome segregation were enriched in mRNAs with decreased m^6^A peaks (Fig. 2E).

GO analysis for transcripts showing increased m^6^A in M1 compared to Mϕ cells revealed a compelling enrichment for biological processes underlying pro-inflammatory responses, including the NF-kappa B signalling pathway, response to lipopolysaccharide and virus, pattern recognition and toll-like receptor signalling pathways, response to interferon-gamma and tumour necrosis factor and regulation of pro-inflammatory cytokines [71–74] (Fig. S2A). Transcripts associated with increased or decreased m^6^A during M2 polarisation were enriched in GO terms associated with homeostatic and anti-inflammatory functions, including tissue development, glucosaminoglycan metabolic processes, regulation of autophagy and vacuolar transport [75–78] (Fig. S2B). These results indicate that m^6^A RNA methylation may have a central role in the regulation of critical pathways that control macrophage differentiation and polarisation.

Using gene set enrichment analysis (GSEA), we identified 143 overlapping gene sets representing transcripts with increased m^6^A, expression and translation levels in Mϕ compared to Mo, and 102 overlapping gene sets showing the opposite pattern (Fig. 2F). In line with the GO analyses, the inflammatory response gene set was significantly enriched in Mϕ compared to Mo, whereas the cell cycle regulation gene set showed significant negative enrichment (Fig. 2G). 153 gene sets representing transcripts with increased m^6^A, expression and translation levels were enriched in M1 compared to Mϕ, including sets associated with an inflammatory response induced by LPS and IFNγ (Fig. S2C and S2D). Only two gene sets were enriched for transcripts with decreased m^6^A, expression and translation levels; one is also associated with downregulation of the inflammatory response (Fig. S2C and S2D). When comparing M2 to Mϕ, we found no significantly enriched gene set in association with decreased m^6^A, gene expression or translation levels. However, 28 gene sets, including sets enriched for immune response and response to IL-4 stimulation genes, typical of M2 cells, were positively associated with increased m^6^A, mRNA expression and translation levels (Fig. S2E and S2F).

Collectively, our in silico analyses using multiple approaches suggest that m^6^A may regulate transcription and translation of transcripts that are directly involved in establishing the identity and function of macrophages.

### m^6^A marks transcripts with critical functions during macrophage differentiation and polarisation

**Fig. 3:**
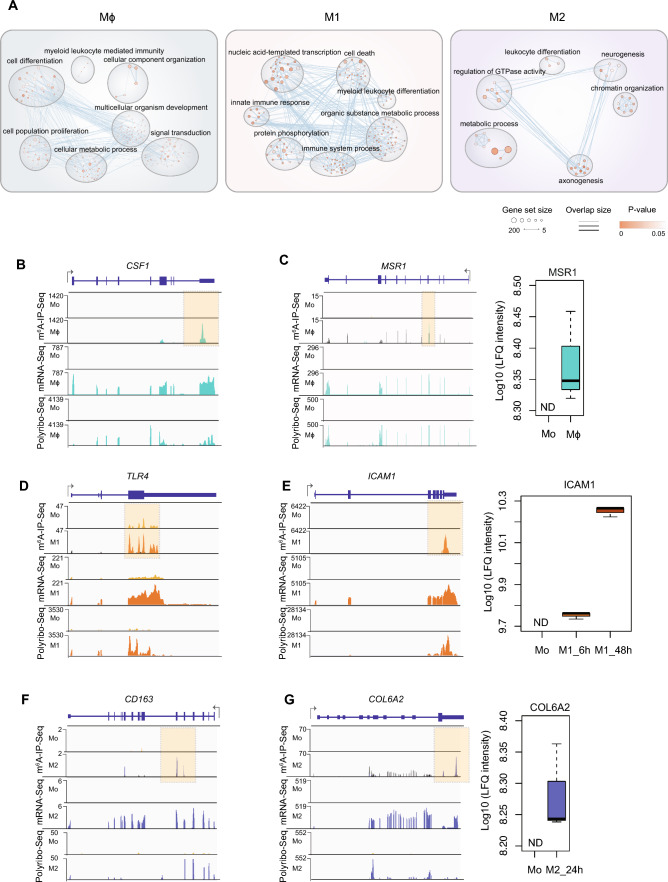
m^6^A marks critical genes that regulate macrophage differentiation and function. **A** Enrichment map of gene ontology terms representing genes with enriched m^6^A peaks in Mϕ, M1 and M2 populations. **B–G** Coverage plots of m^6^A-IP-Seq (top), mRNA-Seq (middle) and Polyribo-Seq (bottom) for **B** *CSF1* and **C** *MSR1* in Mϕ, **D** *TLR4* and **E** *ICAM1* in M1, and **F** *CD163* and **G** *COL6A2* in M2. m^6^A-IP-Seq tracks show the overlay of input (grey) and IP (yellow, green, orange and blue for Mo, Mϕ, M1 and M2 respectively) data. m^6^A peaks are highlighted within a dotted box. m^6^A-IP-Seq coverage plots are displayed in BPM (bins per million reads, Bin size = 1). mRNA-Seq and Polyribo-Seq coverage plots are displayed in RPKM (reads per kilobase per million reads). **C**,** E** and** G** Right: Protein abundance as measured by LC–MS/MS, ND, non-detected. Peptides corresponding to CSF1, TLR4 and CD163 are not detectable by LC–MS/MS, potentially due to the common limitations of mass spectrometry including low signal-to-noise events, loss of spectra due to unanticipated protease digestion problems and unexpected post-translational modifications

Coupling of m^6^A with transcriptional and translational changes occurs on critical genes involved in different states of macrophages (Figs. 2D, 3A and Table S3), indicating that m^6^A may be a regulatory mechanism controlling their expression and function. m^6^A was present on transcripts with essential roles in macrophage development, maintenance and phagocytic function, including *CSF1* (Macrophage Colony Stimulating factor 1) [79], *MSR1* (Macrophage Scavenger Receptor 1) [80] and *CD36* (Scavenger Receptor Class B, Member 3) [81], in association with increased gene expression and translation levels in macrophages compared to monocytes (Fig. 3B, 3C and S3A). In M1 pro-inflammatory-like macrophages, m^6^A was found in transcripts that are highly expressed and translated during macrophage response to infection including *TLR4* (Toll-Like Receptor 4) [82], *ICAM1* (Intracellular Adhesion Molecule 1) [83] and *YAP1* (Yes-associated protein 1) [84] (Figs. 3D, 3E and S3B). In M2 macrophages, the scavenger receptor *CD163*, the anti-inflammatory factor *COL6A2* (Collagen type VI alpha 2 chain), and the C-type lectin *CD209* were all m^6^A-modified (Figs. 3F, G and S3C). These genes have established roles in M2 macrophage biology [85–87]. In conclusion, we found that m^6^A is present in genes that are pivotal for macrophage functions.

### Transcriptome-wide RNA 5 hmC profiling reveals its presence on transcripts relevant to macrophage differentiation and function

**Fig. 4.**
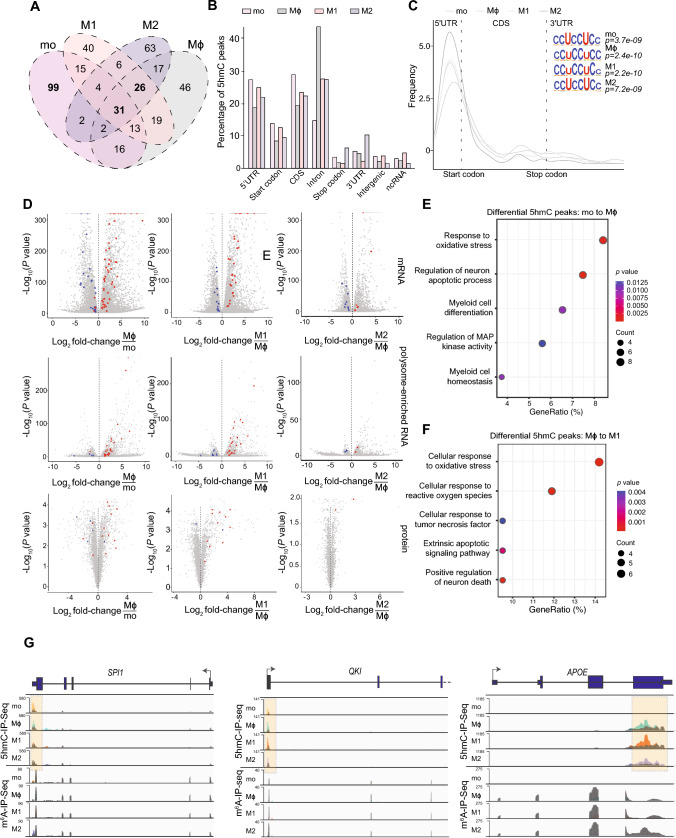
5 hmC is present in genes involved in monocyte-to-macrophage differentiation and polarisation. **A** Venn diagram showing the overlap of 5 hmC peaks detected in Mo, Mϕ, M1 and M2. **B** Percentage of 5 hmC peaks identified by 5 hmC-IP-Seq in defined transcript and genomic regions: coding sequence (CDS), 5′ untranslated region (5′UTR), 3′ untranslated region (3′UTR), start and stop codons, intergenic regions and non-coding RNA (ncRNA) found in Mo, Mϕ, M1 and M2. **C** Metagene profiles detailing 5 hmC distribution along a normalised transcript and top enriched sequence motifs identified by 5 hmC-IP-Seq in all cell types. **D** Volcano plots showing differential gene expression (mRNA, top), translation (polysome-enriched RNA, middle) and protein (bottom) levels. Differential 5 hmC peaks (red: increased, blue: decreased) are shown for Mϕ compared to Mo, M1 compared to Mϕ and M2 compared to Mϕ. **E** Gene ontology analysis on differential 5 hmC peaks during Mo to Mϕ differentiation **F** Coverage plots for 5 hmC-IP-Seq (top) and m^6^A-IP-Seq (bottom) data from Mo, Mϕ, M1 and M2 shown for *SPI*, *QKI1* and *APOE*. 5 hmC- and m^6^A-IP-Seq tracks show the overlay of input (grey) and IP (yellow, green, orange and purple for Mo, Mϕ, M1 and M2 respectively) data. A 5 hmC peak is highlighted within a dotted box. 5 hmC and m^6^A-IP-Seq coverage plots are displayed in BPM (bins per million reads, Bin size = 1). **G** Gene ontology analysis on differential 5 hmC peaks during Mϕ to M1 polarisation

Next, we examined the transcriptome-wide distribution of 5 hmC in monocytes and macrophages using 5 hmC RNA immunoprecipitation sequencing (5 hmC-IP-Seq). Overall, we identified between 151 and 182 5 hmC peaks for monocytes and macrophages corresponding to 128–161 genes (Table S4), of which 31 5 hmC peaks (corresponding to 24 genes) were common to monocytes and macrophages, 99 5 hmC peaks (in 94 genes) were exclusive to monocytes and 26 5 hmC peaks (in 25 genes) were shared by all macrophages (Fig. 4A). Despite a global decrease of RNA 5 hmC in macrophages compared to monocytes (Fig. 1E and G), the number of increased and decreased RNA 5 hmC peaks in Mϕ compared to Mo was similar (Fig. S4A). The number of differential 5 hmC peaks detected during the transition of Mϕ to M1 or Mϕ to M2 was less than half of those observed in Mo to Mϕ (Fig. 4A). This finding is consistent with the observed lack of changes in global 5 hmC levels between polarised and non-polarised macrophages (Fig. 1E and G).

The majority of 5 hmC peaks were found in the CDS, intronic and 5′ UTR regions (~ 25% for each feature) (Fig. 4B). In contrast to m^6^A, 5 hmC metagene profiles revealed strong enrichment of this modification towards the 5′ UTR of mRNA transcripts (Fig. 4C). In addition to an enrichment of UCC-rich repeats reported previously in peaks containing RNA 5 hmC [21, 23], we also identified CCG- and CA-rich motifs in all four stages of monocyte-to-macrophage differentiation and polarisation (Figs. 4C and S4B). Notably, like m^6^A, we also observed a trend by which RNA 5 hmC levels are enriched in highly expressed and translated transcripts as Mo differentiate into Mϕ and following polarisation of Mϕ into M1 macrophages (Fig. 4D). The association between RNA 5 hmC levels and transcription or translation during M2 polarisation was not as obvious, possibly due to fewer quantifiable transcripts and proteins in M2 macrophages (Fig. 4D).

GO analysis on transcripts with differential RNA 5 hmC peaks between Mo and Mϕ revealed enrichment of functions relevant to myeloid differentiation and homeostasis (Fig. 4E). Relevant genes within these GO categories include the macrophage transcription factor *SPI1*/*PU.1* [88], and *FOXO3* which regulates autophagy [89]; an essential stimulus for monocyte-macrophage differentiation (Figs. 4F and S4C). *QKI* transcripts encoding a splicing factor that promotes monocyte differentiation into macrophages also exhibited increased RNA 5 hmC peak in Mϕ [90] (Fig. 4F). Functions associated with the regulation of MAP kinase activity and oxidative stress, which are pivotal for macrophage differentiation [91, 92], were enriched (Fig. 4E). Significant enrichment of differential RNA 5 hmC peaks was also observed in *MCL1,* a switch that modulates macrophage viability or apoptosis during bacterial clearance [93] (Fig. S4D).

In line with the roles of M1 macrophages, differential RNA 5 hmC peaks in M1 compared to Mϕ were enriched in functions associated with responses to reactive oxygen species, tumour necrosis factor and apoptosis involved in pro-inflammatory processes [73, 92, 93] (Fig. 4G). Key genes within these categories include *APOE* and *MCL1* [93, 94] (Figs. 4F and S4D). GO analysis was not performed on M2 macrophages given the limited number of differential RNA 5 hmC peaks (n = 30) in M2 compared to Mϕ. However, we observed enriched RNA 5 hmC peaks in transcripts encoding proteins known to be expressed at high levels and/or secreted by M2. They include a classical M2 marker, FN1 (44), and a mesenchymal marker, Vimentin (VIM) (Fig. S4E); both have been shown to be involved in the anti-inflammatory process of wound healing [95, 96]. In summary, we report that RNA 5 hmC is present in transcripts with functional relevance in macrophages.

### RNA m^6^A and 5 hmC co-occur on opposite ends of key monocyte and macrophage transcripts

**Fig. 5.**
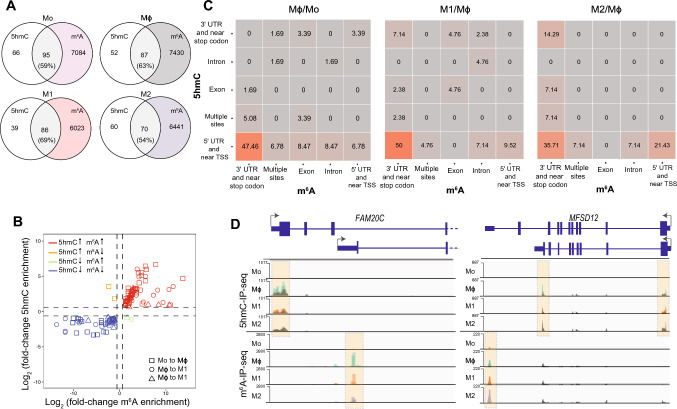
5 hmC and m^6^A co-occur in genes that regulate macrophage differentiation and polarisation. **A** Number and percentages of transcripts bearing both m^6^A and 5 hmC in Mo, Mϕ, M1 and M2. **B** The association between m^6^A and 5 hmC enrichment on 5 hmC-modified transcripts during macrophage differentiation and polarisation. **C** The proportion of differentially enriched 5 hmC and m^6^A peaks that co-occurred in defined transcript regions (UTR, untranslated region; TSS, transcriptional start site, exon and intron) during macrophage differentiation and polarisation. **D** 5 hmC and m^6^A peaks in *FAM20C* and *MSDF12* transcripts identified in 5 hmC-IP-Seq (top) and m^6^A-IP-Seq (bottom) data from Mo, Mϕ, M1 and M2. 5 hmC- and m^6^A-IP-Seq tracks show the overlay of input and IP data. m^6^A and 5 hmC peaks are highlighted within dotted boxes. 5 hmC- and m^6^A-IP-Seq coverage plots are displayed in BPM (bins per million reads, Bin size =1)

Given that m^6^A and 5 hmC-modified mRNAs were both enriched for functions pertinent to different states of macrophages, we determined whether these modifications occurred on the same transcript or if the presence of one or the other was exclusive. Nearly two-thirds of the mRNAs bearing 5 hmC in monocytes or macrophages were also modified by m^6^A (Fig. 5A). A significant association between 5 hmC and m^6^A changes was also observed during monocyte-to-macrophage differentiation and polarisation (Fig. 5B, p = 2.2e−16, Fisher’s Exact Test). mRNAs bearing both 5 hmC and m^6^A marks include some of those highlighted above: *FOXO3* and *MCL1* (Fig. S4C and S4D). However, we did not detect m^6^A peaks in the other 5 hmC-modified transcripts described herein (Figs. 4F and S4E). In the vast majority of transcripts, RNA 5 hmC peaks were found in the 5' UTR and near transcriptional start sites, whereas m^6^A peaks were enriched in the 3' UTR and near stop codons (Fig. 5C). Notably, in some cases, such as *FAM20C* and *MFSD12*, we observed an enrichment of 5 hmC and m^6^A peaks mapping to different alternative 5' and 3' isoforms in macrophages (Fig. 5D).

Overall, we have identified several key genes presenting differential 5 hmC methylation in monocytes and macrophages. As with m^6^A, we observe an enrichment of 5 hmC in genes that are highly expressed and translated in these cells, suggesting that 5 hmC may also be regulating genes with functions relevant to monocytes and macrophages. Furthermore, we identify important macrophage regulators bearing both RNA modifications, located on opposite ends of the mRNA. To the best of our knowledge, these results are the first to identify the presence of 5 hmC in mRNAs that are also m^6^A-modified. The co-occurrence of these modifications on these genes adds another dimension to the complex regulatory dynamics exhibited by RNA.

### METTL3 regulates the expression of macrophage differentiation and polarisation genes

**Fig. 6:**
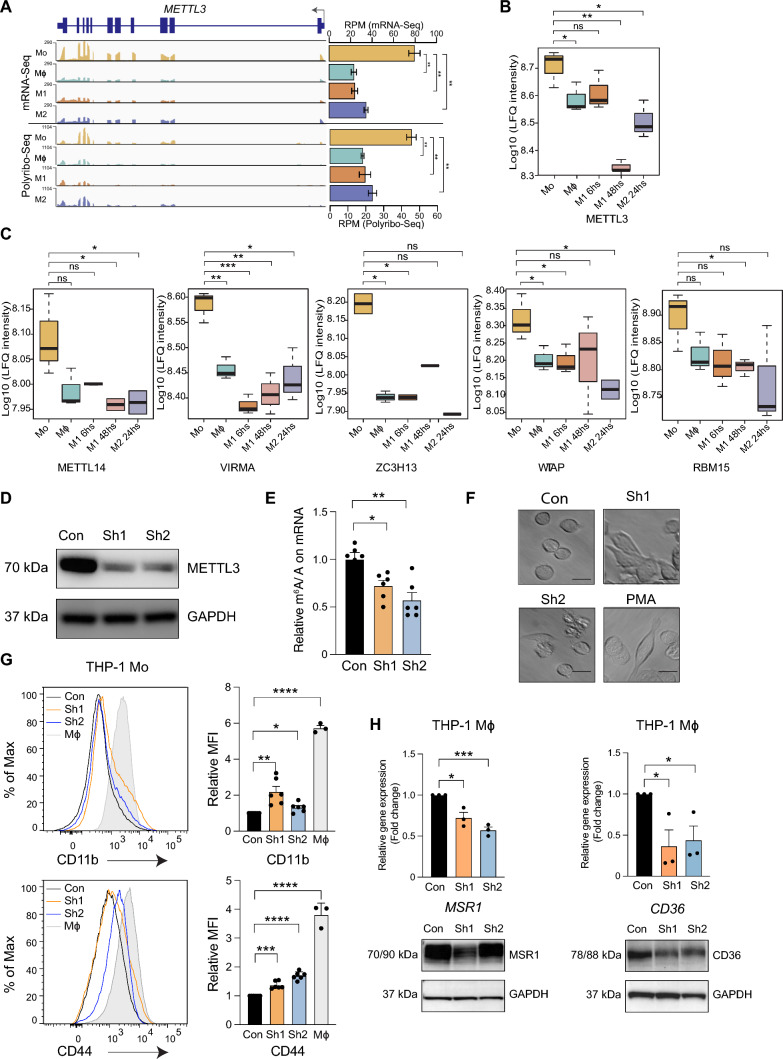
Stable METTL3 depletion may inadvertently perturb gene expression necessary for monocyte and macrophage differentiation and polarisation. **A** *METTL3* coverage plots showing reads per kilobase per million reads (RPKM) for mRNA-Seq (top) and Polyribo-Seq (bottom) data from Mo, Mϕ, M1 and M2. **B** METTL3 protein abundance as measured by LC–MS/MS. **C** METTL14, VIRMA, ZC3H13, WTAP and RBM15 protein levels in Mo, Mϕ, M1 and M2 as measured by LC–MS/MS. **D** Western blot showing METTL3 and GAPDH protein levels following THP-1 transduction with control (Con) or METTL3-specific (Sh1 and Sh2) shRNAs. **E** Relative m^6^A/A in polyadenylated RNA from control and METTL3 depleted cells measured by LC–MS/MS. **F** Microscopy images showing morphological changes in THP-1 cells transduced with control and METTL3-targeting shRNAs or exposed to PMA (positive control). **G** Flow cytometry profiles and quantification (Mean Fluorescence Intensity (MFI) showing changes in the relative expression of the macrophage cell surface markers CD11b and CD44 following METTL3 depletion in Mo. **H** Gene expression (top) and protein level (bottom) changes for MSR1 and CD36 following METTL3 depletion in Mϕ cells. All data are from at least three independent experiments. Bar plots show mean ± SEM. An unpaired two-tailed Student’s t-test was used to determine significance, denoted by ns, not significant; *, *p* < 0.05; *** and *p* < 0.001

We determined the expression of m^6^A regulatory genes involved in macrophage differentiation and observed a significant decrease in *METTL3* expression by mRNA-seq in macrophages compared to monocytes (Fig. 6A). A relatively lower abundance of METTL3 transcripts was detected in the polysome fractions of macrophages compared to monocytes (Fig. 6A). Protein levels of METTL3 and other components of the m^6^A writer complex (METTL14, VIRMA, ZC3H13, WTAP and RBM15) were also diminished in macrophages compared to monocytes (Fig. 6B and C). This decrease in protein levels of components of the methyltransferase complex, including METTL3 that deposits m^6^A, explains the observed decrease of global m^6^A levels during monocyte-to-macrophage differentiation (Fig. 1D).

To validate the role of METLL3 and m^6^A during monocyte-to-macrophage differentiation in vitro*,* we depleted *METTL3* in THP-1 (Mo) cells using short hairpin RNAs (Fig. 6D). As expected, the depletion of *METTL3* led to a significant decrease in global m^6^A levels on mRNA (Fig. 6E). Following METTL3 depletion, THP-1 cells (Mo) exhibited macrophage-like morphology, including adherence to the bottom of the culture flask, enlargement of the cell body and amoeboid appearance (Fig. 6F). Additionally, METTL3-depleted cells expressed higher levels of the macrophage-associated cell surface markers that are non-m6A-modified including CD11b and CD44 (Fig. 6G). However, their expression levels were markedly lower (3- to 4-fold less in mean fluorescence intensity) than PMA-differentiated cells, indicating that the spontaneous differentiation into macrophages facilitated by METTL3 depletion was incomplete. Notably, in METTL3-depleted Mϕ macrophages (Fig. 6D), which demonstrated significantly lower global m^6^A levels on mRNA (Fig. 6E), we observed a significant reduction in both mRNA expression and protein levels of MSR1 (Fig. 6H) and CD36 (Fig. 6I), and mRNA expression of *CSF1* (Fig. S5A). Therefore, stable depletion of METTL3 may have caused diminished m^6^A deposition and consequently diminished expression of these genes, which would otherwise increase during macrophage differentiation (Figs. 3B, C and S3A). We further confirmed in M1 and M2 macrophages that engineered METTL3 depletion led to reduced mRNA and/or protein levels encoded by key genes that should normally be more highly expressed in these macrophage subtypes (Figs. 3D–G, S3B, S3C, S5B and S5C). Notably, for *ICAM1* and *YAP1*, METTL3 depletion did not consistently alter the expression of *ICAM1* and *YAP1* mRNAs but decreased ICAM1 and YAP1 protein levels, consistent with previous reports that m^6^A changes may directly affect protein translation without altering mRNA levels [97, 98]. Collectively, our results indicate that the persistence of m^6^A is required to regulate key macrophage differentiation and polarisation genes despite a global METTL3 loss in macrophages. Stable depletion of METTL3 alone without profiling how m^6^A changes alters the expression of key genes involved during progressive macrophage differentiation and polarisation may mask the identification of critical genes in these processes that may be regulated via m^6^A.

### Regulation of RNA 5 hmC by TET enzymes controls the stability of mRNA in macrophages

**Fig. 7:**
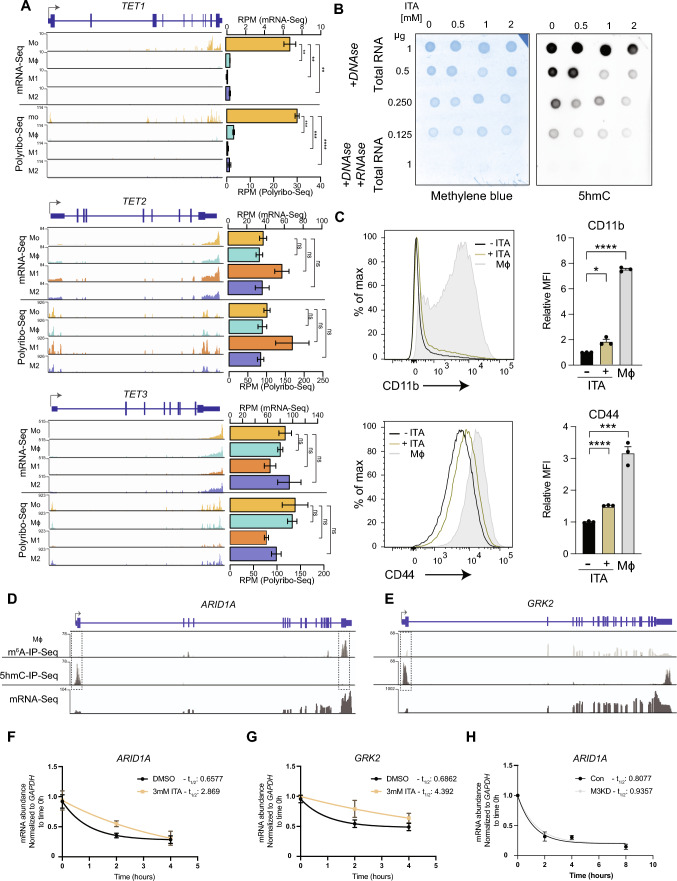
TET-regulated RNA 5 hmC controls half-life of critical transcripts in macrophages. **A** *TET1,2 and 3* coverage plots showing reads (RPKM) for mRNA-Seq (top) and Polyribo-Seq (bottom) data from Mo, Mϕ, M1 and M2. **B** Dot blot on total RNA from THP-1 Mo treated with increasing concentrations of Itaconic Acid (ITA) probed with anti-5 hmC antibody (right) and methylene blue loading control (left). Samples were treated with DNase to eliminate the possibility of detecting 5 hmC on DNA. Negative controls were treated with both DNase and RNase A. **C** Flow cytometry profiles and quantification (Mean Fluorescence Intensity (MFI)) showing changes in the relative expression of Mϕ cell surface markers CD11b and CD44, following treatment of THP-1 Mo with 2 mM Itaconic Acid (ITA) compared to control. Coverage plots of m^6^A-IP-Seq (top), 5 hmC-IP-Seq (middle) and mRNA-Seq (bottom) data for **D** *ARID1A* and **E** *GRK2* in Mϕ. **F **and** G** mRNA decay plots for *ARID1A* and *GRK2* in Itaconic Acid (ITA)-treated Mϕ. **H** mRNA decay plots for *ARID1A in* METTL3-depleted (M3KD) Mϕ**.** All data are from at least three independent experiments. Bar plots show mean ± SEM. An unpaired two-tailed Student’s t-test was used to determine significance, denoted by *, *p* < 0.05; ***, *p* < 0.001 and ****, *p* < 0.0001

Given that TET enzymes are known to regulate RNA 5 hmC, we determined whether their expression is also associated with the decrease of RNA 5 hmC during monocyte-to-macrophage differentiation. Based on mRNA-Seq data, *TET1* expression diminished during monocyte-to-macrophage differentiation and our polysome profiling data similarly inferred lower *TET1* translation in macrophages compared to monocytes (Fig. 7A). *TET2* and *TET3* mRNA enrichment showed no significant differences in the polysome fraction of macrophages compared to monocytes (Fig. 7A). Collectively, these data demonstrate that TET1, but not TET2 or TET3, expression correlated best with global RNA 5 hmC levels. We therefore speculate that TET1 may be the primary mediator of RNA 5-hydroxymethylation during monocyte-to-macrophage differentiation.

Given that there is no reliable antibody against TET1 to validate the depletion of TET1 in cells, we resorted to inhibiting the catalytic activity of TETs by using the metabolite Itaconic Acid (ITA) to block their active sites, as reported in our recent study [99]. We confirmed that inhibition of TETs’ activities led to a decrease in global RNA 5 hmC levels in THP-1 monocytes (Fig. 7B). Following ITA treatment, THP-1 monocytes spontaneously expressed higher levels of the macrophage markers CD11b and CD44, although these changes were markedly lower than the levels achieved via PMA treatment (Fig. 7C). Our data indicate that inhibition of TETs in THP-1 monocytes may trigger their differentiation into macrophages but, like METTL3 depletion, it is insufficient to facilitate complete differentiation and polarisation of macrophages.

While exploring our 5 hmC-IP-Seq data, two genes were particularly noteworthy: *ARID1A* and *GRK2* (Fig. 7D and 7E). *ARID1A* (AT-Rich Interaction Domain 1A), a principal component of the BAF SWI/SNF chromatin remodelling complex, is required for maintenance of lineage-specific enhancers [100]**,** essential for myeloid differentiation [101] and induces the antiviral interferon response in macrophages [102]. *GRK2* (G Protein-Coupled Receptor Kinase 2), a central signalling node that modulates G protein-coupled receptors (GPCRs) and other cell signalling routes including the NF-KB and MAPK inflammatory pathways, is a critical regulator of chemotaxis [103] and myeloid-specific deficiency of *GRK2* results in excessive cytokine production in a sepsis model [104]. Since both *ARID1A* and *GRK2* have important functions in myeloid cell fate, we reasoned that the expression of these genes must be tightly regulated and hypothesised that RNA 5 hmC is involved in this process. Even though the role of RNA 5 hmC is still unclear, studies have linked the presence of this mark with transcript stability [23, 25]. We therefore determined whether 5 hmC regulates the stability of *ARID1A* and *GRK2* transcripts by measuring their decay rates in 5 hmC-depleted Mϕ using ITA to block TETs’ activity. As a negative control *MYC* transcripts are not modified by 5 hmC (Fig. S6A) and thus, *MYC* RNA half-life was not affected by ITA treatment in Mϕ (Fig. S6B). However, the half-life of *ARID1A* and *GRK2* was 4 to 6 times longer in 5 hmC-depleted Mϕ (Fig. 7F and G). This finding is consistent with previous work performed in mESCs showing that RNA 5 hmC reduces the stability of crucial pluripotency-promoting transcripts (27) and that 5 hmC promotes *MERVL* retrotransposon destabilization (30). Given that *ARID1A* but not *GRK2* (Fig. 7D and E) harbours m^6^A, we also measured *ARID1A* RNA decay rate following METTL3 depletion in Mϕ (Fig. 7H) and found that METTL3 depletion does not affect *ARID1A* half-life. These data suggest that TET-mediated RNA 5 hmC reduces the stability of *ARID1A* and *GRK2* in THP-1- derived macrophages independently of m^6^A.

## Discussion

Following infection, sensing of danger signals leads to activation of the innate immune response and profound phenotypic changes drive monocyte-to-macrophage differentiation and polarisation [29, 31, 78]. Due to the critical role of these immune cells, extensive research has been conducted to understand the cellular and molecular mechanisms controlling them in homeostasis and disease. However, little is known as to how RNA modifications and altered expression of RNA modification enzymes are involved in this process. To address this gap, we generated a multi-omics dataset that profiled the transcriptome, m^6^A and 5 hmC epitranscriptomes, polyribosome-enriched mRNAs and proteome across four monocyte-macrophage states (Fig. 1A) and analysed m^6^A and 5 hmC patterns in the context of monocyte-to-macrophage differentiation and polarisation.

Despite an overall decrease in RNA m^6^A and 5 hmC levels in macrophages compared to monocytes (Fig. 1D, E and G), these modifications are present on a substantial number of transcripts (Figs. 2A and 4A). This observation implies that a global decrease of a particular RNA modification does not mean that all transcripts should also be ‘less’ modified and therefore, carry the functional consequences of the absence or a decrease of specific RNA modification. For example, while transcripts associated with the cessation of cell differentiation lose m^6^A, others that are critical for cell differentiation gained m^6^A to maintain the fidelity of monocyte-to-macrophage differentiation. Consistent with the need for RNA modifications to persist during cellular differentiation to maintain and/or enhance the levels of transcripts that are critical for this process, we observed incomplete monocyte-to-macrophage differentiation following METTL3 depletion or 5 hmC inhibition. Forceful removal of m^6^A and 5 hmC may inadvertently remove modifications that are required for the expression of specific transcripts to enhance this process. As such, this common experimental approach may not recapitulate the context-specific re-distribution of RNA modifications on defined sets of transcripts to define cellular identity. Our observations highlight the importance of mapping RNA modifications during progressive stages of cell differentiation rather than assessing RNA modification following the depletion of specific regulatory proteins to elucidate dynamic changes. In addition, some transcripts bearing RNA modifications may not promote monocyte differentiation and macrophage polarisation per se but are important to regulate macrophage functions when encountering specific environmental cues. Herein, we have provided key examples of these genes, but many others remain to be identified in future studies.

Through an integrative approach, we have identified stage-specific enrichment of RNA m^6^A and 5 hmC on select monocyte-macrophage genes (Figs. 2D and 4D), coherent with previously proposed co-transcriptional deposition models where m^6^A [67, 68] and 5 hmC [23] are deposited as transcription proceeds. In line with our findings, m^6^A and 5 hmC have been shown to promote translation in different contexts [21, 70, 105]. Interestingly, while RNA 5 hmC abundance is positively correlated with active mRNA translation in *D.melanogaster* [21], it did not seem to impact mRNA translation but instead reduced the stability of critical transcripts in mESCs [23]. By focusing on *ARID1A* and *GRK2*, our results support the role of 5 hmC in regulating transcript stability in mammalian cells (Fig. 7F and H). Since the temporal expression order of inflammatory molecules is influenced by mRNA stability (113), 5 hmC may be an additional regulatory layer that fine-tunes gene expression timing of key genes during the inflammatory response.

Another noteworthy observation arising from our study is that transcripts harbouring 5 hmC often harbour m^6^A, frequently on opposite ends of the molecule (Fig. 5C). In some cases, 5 hmC and m^6^A mark alternatively spliced isoforms (Fig. 5D), indicating that they may regulate alternative splicing and/or functions of these specific isoforms. Recently, others have recognised the interplay between RNA modifications [106]. For example, cooperation between m^6^A and 5mC has been reported to promote replication of murine leukemia virus [107] and enhance p21 translation in oxidative stress-induced cellular senescence [108]. Since RNA 5 hmC is a product of 5mC oxidation [109], it is tempting to speculate that an m^6^A-5mC-5 hmC axis can act on specific transcripts under distinct cellular contexts. Future studies exploring the nature of the interaction between RNA modifications should aim to establish whether one modification prevails over others and if they act synergistically or exclusively. Herein, we provide evidence that RNA 5 hmC can promote mRNA decay independently of m^6^A (Fig. 7F and H).

While there are studies investigating TET enzymes and DNA 5 hmC in macrophages [110–112], their roles in gene expression regulation remain unclear. To advance the current understanding of these enzymes in macrophage biology, their contribution to both RNA and DNA 5 hmC must be considered. Future research should aim to establish the mechanisms underpinning TETs' selectivity towards DNA and/or RNA. It is plausible that crosstalk between RNA-m^6^A and DNA 5 hmC is also involved in macrophage biology. Notably, recent work has shown that RNA-m^6^A regulates TET-1-mediated DNA 5 hmC to promote transcription and chromatin accessibility in cancer [113]. Future work should aim to distinguish the importance of the RNA-m^6^A-RNA-5 hmC axis and the RNA-m^6^A-DNA-5 hmC axis to gene expression regulation in macrophage development and functions. Our observations also remain to be confirmed in primary human monocytes and macrophages at specific stages of differentiation and polarization under physiological and pathological conditions.

Altogether, our observations raise at least three important points; first, patterns/associations observed at the global level cannot be directly extrapolated to explain mechanisms acting at the transcript level. Second, these data reinforce a concept that has emerged in the m^6^A literature [114], namely that RNA modifications have context-specific functions, and this is likely to be dictated by context-specific expression patterns of writers, erasers and reader proteins. As opposed to naturally occurring changes in the expression of these proteins that leads to redistribution of RNA modification across the transcriptome, stable depletion of one or more of them is likely to perturb the expression of key genes on which RNA modification needs to persist to regulate a given physiological process. Hence, experiments designed to profile RNA modifications alongside mRNA and protein changes during progressive states of cellular differentiation and polarisation is essential to identify key genes involved in these processes. Third, a transcript’s fate could be determined by more than one RNA modification, several modifications could co-exist and perhaps interact, cooperate and/or cross-regulate in different biological contexts.

From a clinical perspective, macrophage dysfunction has been associated with the development of multiple human disorders. In the context of cancer, tumour-associated macrophages derived from bone marrow monocytes are recruited through inflammatory signals released by cancer cells to enhance tumour progression [115]. This class of macrophages also promotes tumour relapses from standard therapies via maintenance of stem cells, promotion of angiogenesis and perturbation of immune response [116–118]. Macrophages are involved in facilitating the thickening of arteries consequent to accumulation of plaques around the artery wall, leading to atherosclerosis [119]. Both pro- and anti-inflammatory macrophages can contribute to the pathogenesis and progression of common autoimmune disorders such as rheumatoid arthritis, and inflammatory diseases including osteoarthritis and diabetes [120–122]. For these reasons, macrophage reprogramming has emerged as an attractive approach for therapy. By adding to the current understanding of how the epitranscriptome influences macrophage plasticity, our work provides a novel perspective and resource that may inform new strategies to reprogram macrophages for the treatment of human diseases. These strategies should not only consider the use of global RNA m^6^A or 5 hmC modulators such as METTL3 or TET inhibitors as they may inadvertently remove RNA modification marks that are essential for complete macrophage differentiation. As such, it would be critical to identify specific macrophage-associated genes that were affected by abnormal RNA methylation in diseases and develop specific RNA methylation editing tools, including CRISPR-dCas13-based RNA editing approaches [123, 124], to rectify these abnormalities. Our work also indicates the importance of understanding the interplay between multiple RNA modifications in diseases to design the best therapeutic approaches.

### Supplementary Information

Below is the link to the electronic supplementary material.

Figure S1. Validation of macrophage differentiation and polarisation. Flow cytometry profiles showing expression levels of cell surface markers in (A) differentiated and (B) polarised macrophages. Relative expression of differentiated (C) and polarised (D) macrophage markers measured by qPCR. Principal Component Analysis (PCA) of (E) RNA-Seq and (F) LC-MS/MS data from Mo, Mϕ, M1_6h, M1_48h and M2. All data are from at least three independent experiments and show mean ± SEM. An unpaired two-tailed Student’s t-test was used to determine significance, denoted by ns, not significant; *, *p* <0.05; **, *p* <0.01, ***, *p* <0.001 and ****, *p* <0.0001. Supplementary file1 (PDF 767 KB)

Figure S2. Association between m^6^A changes and enrichment of gene functions relevant to polarised macrophages. Gene ontology analysis on increased (top) and decreased (bottom) m^6^A peaks during (A) Mϕ to M1 and (B) Mϕ to M2 polarisation. (C) Venn diagram showing overlapping gene sets identified by Gene Set Enrichment Analysis (GSEA) on m^6^A-IP-Seq, mRNA-Seq and Polyribo-Seq data presenting positive (top) or negative (bottom) enrichment during Mϕ to M1. (D) GSEA signatures showing significantly positive and negative enrichment during Mϕ to M1 polarisation. (E) Similar Venn diagram shown in (C) for Mϕ to M2 polarisation. (F) GSEA signature showing significantly positive enrichment during Mϕ to M2 polarisation. Supplementary file2 (PDF 1888 KB)

Figure S3. m^6^A is present in genes that regulate macrophage differentiation and function. (A-C) Left: Coverage plots of m^6^A-IP-Seq (top), mRNA-Seq (middle) and Polyribo-Seq (bottom) for (A) CD36 in Mϕ, (B) YAP1 in M1 and (C) CD209 and in M2. m^6^A-IP-Seq tracks show the overlay of input (grey) and IP (yellow, green, orange and blue for Mo, Mϕ, M1 and M2 respectively) data. An m^6^A peak is highlighted within a dotted box. m^6^A-IP-Seq coverage plots are displayed in BPM (bins per million reads, Bin size=1). mRNA-Seq and Polyribo-Seq coverage plots are displayed in RPKM (reads per kilobase per million reads). Right: Protein abundance as measured by LC-MS/MS, ND, non-detected. Peptides corresponding to YAP1 were not detected by LC-MS/MS. Supplementary file3 (PDF 531 KB)

Figure S4. Co-occurence of 5 hmC and m^6^A in genes that are involved in macrophage differentiation and polarisation. (A) Number of increased (red) and decreased (blue) 5 hmC peaks during macrophage differentiation and polarisation. (B) Significantly enriched sequence motifs identified by 5 hmC-IP-Seq in Mo, Mϕ, M1 and M2. 5 hmC and m^6^A peaks in FOXO3 (C), MCL1 (D), VIM and FN1 (E) transcripts identified in 5 hmC-IP-Seq (top) and m^6^A-IP-Seq (bottom) data from Mo, Mϕ, M1 and M2. 5 hmC- and m^6^A-IP-Seq tracks show the overlay of input and IP data. m^6^A and 5hmC peaks are highlighted within dotted boxes. 5 hmC- and m^6^A-IP-Seq coverage plots are displayed in BPM (bins per million reads, Bin size=1). Supplementary file4 (PDF 620 KB)

Figure S5. Expression of genes relevant to monocyte and macrophage differentiation and polarisation following METTL3 knockdown. Gene expression changes for CSF1 (A), YAP1, ICAM1, TLR4 (B) and CD163, COL6A2 and CD209 (C) following METTL3 depletion in Mϕ, M1 and M2 cells respectively. (B bottom) western blots showing YAP1 and ICAM1 protein levels following THP-1 transduction with control (Con) or METTL3-specific (Sh1 and Sh2) shRNAs. All data are from at least two independent experiments. Bar plots show mean ± SEM. An unpaired two-tailed Student’s t-test was used to determine significance, denoted by ns, not significant; *, *p* <0.05; *** and *p* <0.001. Supplementary file5 (PDF 1062 KB)

Figure S6. Half-life of *MYC *mRNA following treatment with Itaconic Acid. (A) Coverage plots of 5 hmC-IP-Seq (top) and mRNA-Seq (bottom) data for MYC in Mϕ. (B) mRNA decay plot for *MYC* in Itaconic Acid (ITA)-treated Mϕ. Supplementary file6 (PDF 467 KB)

Table S1. Oligonucleotide sequences.Supplementary file7 (XLSX 11 KB)

Table S2. Lists of differentially enriched m^6^A peaks during macrophage differentiation and polarisation. Supplementary file8 (XLSX 956 KB)

Table S3: Lists of gene ontology terms representing genes with enriched m^6^A peaks in Mϕ, M1 and M2 populations used to generate enrichment maps from Figure 3A. Supplementary file9 (XLSX 101 KB)

Table S4: Lists of RNA 5 hmC-enriched peaks in monocytes and macrophages. Supplementary file10 (XLSX 33 KB)

## Data Availability

All data have been deposited at Gene Expression Omnibus (GEO) repository, accession number GSE130011 and GSE213207. Raw MS data have been deposited to the ProteomeXchange Consortium (http://proteomecentral.proteomexchange.org) via the PRIDE partner repository with the dataset identifier (PXD017391).
