# Supplementary figures and images for: Dynamic changes in RNA m6A and 5 hmC influence gene expression programs during macrophage differentiation and polarisation
Source: Cell Mol Life Sci. 2024 May 23;81(1):229. doi: 10.1007/s00018-024-05261-9 (PMC11116364; doi:10.1007/s00018-024-05261-9)

Figure S1

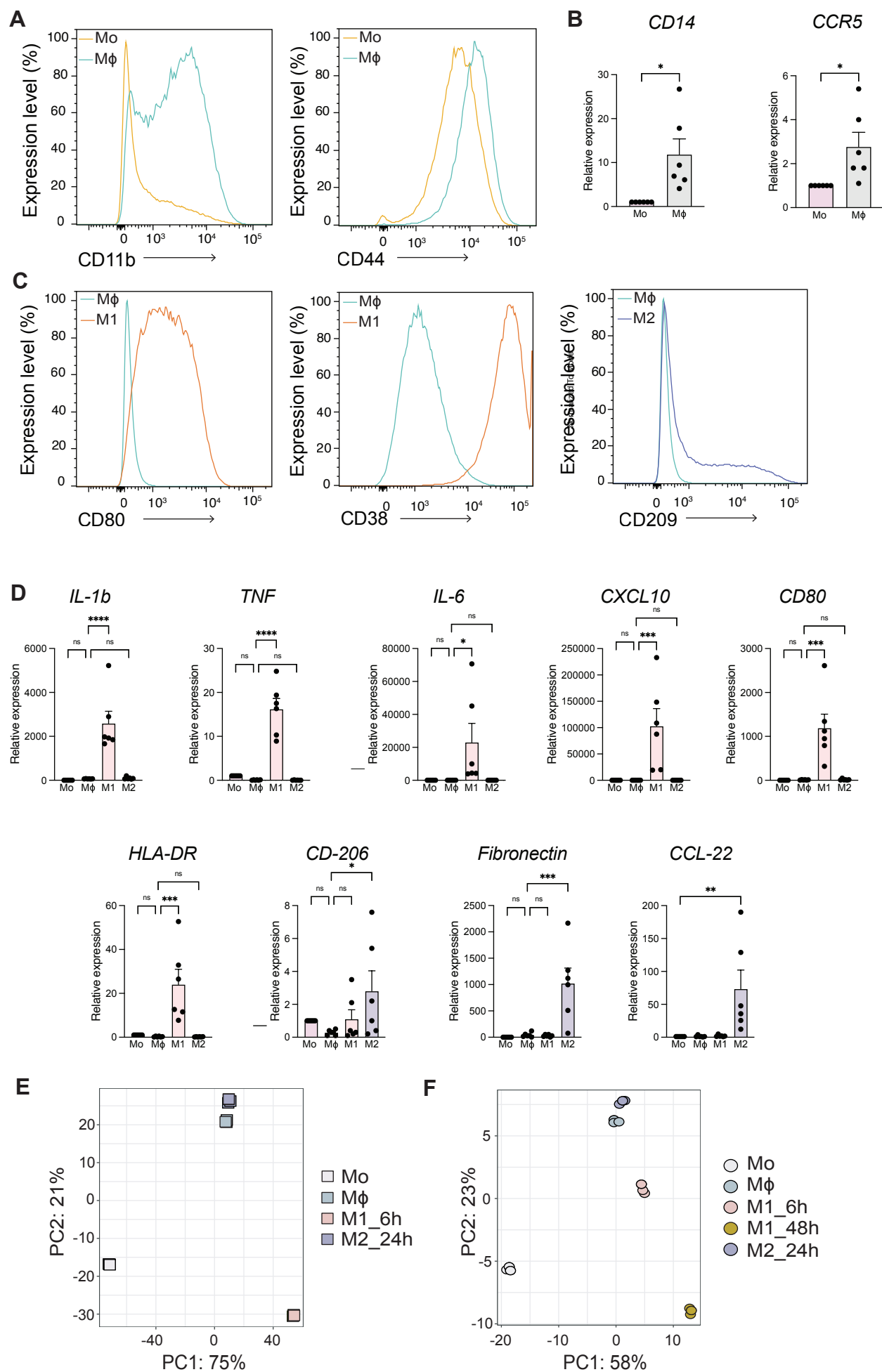

Supplement: Supplementary file 1 — Figure S1. Validation of macrophage differentiation and polarisation. Flow cytometry profiles showing expression levels of cell surface markers in (A) differentiated and (B) polarised macrophages. Relative expression of differentiated (C) and polarised (D) macrophage markers measured by qPCR. Principal Component Analysis (PCA) of (E) RNA-Seq and (F) LC-MS/MS data from Mo, Mϕ, M1_6h, M1_48h and M2. All data are from at least three independent experiments and show mean ± SEM. An unpaired two-tailed Student’s t-test was used to determine significance, denoted by ns, not significant; *, p <0.05; **, p <0.01, ***, p <0.001 and ****, p <0.0001. Supplementary file1 (PDF 767 KB) [file 18_2024_5261_MOESM1_ESM.pdf]

Figure S2

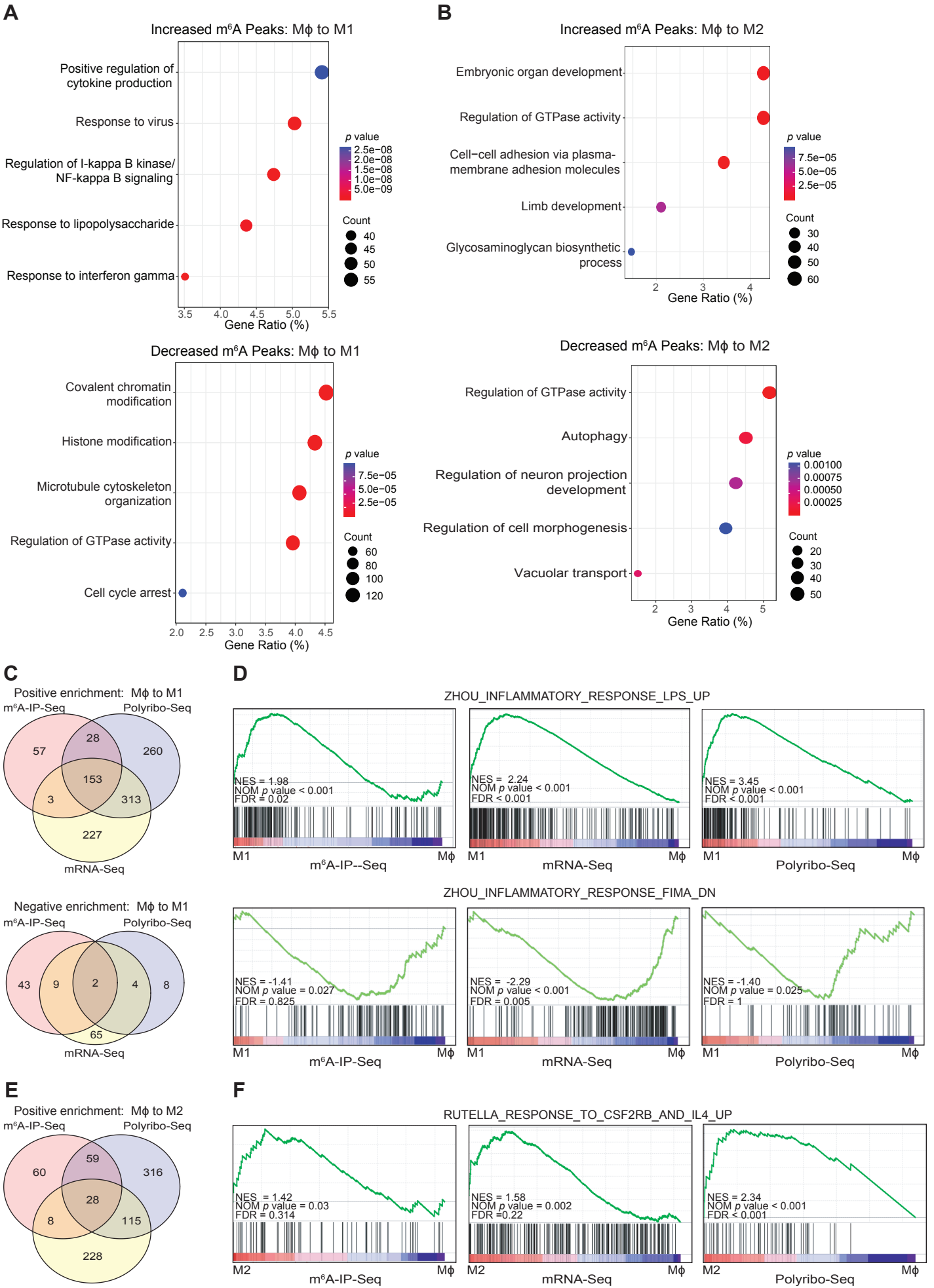

Supplement: Supplementary file 2 — Figure S2. Association between m6A changes and enrichment of gene functions relevant to polarised macrophages. Gene ontology analysis on increased (top) and decreased (bottom) m6A peaks during (A) Mϕ to M1 and (B) Mϕ to M2 polarisation. (C) Venn diagram showing overlapping gene sets identified by Gene Set Enrichment Analysis (GSEA) on m6A-IP-Seq, mRNA-Seq and Polyribo-Seq data presenting positive (top) or negative (bottom) enrichment during Mϕ to M1. (D) GSEA signatures showing significantly positive and negative enrichment during Mϕ to M1 polarisation. (E) Similar Venn diagram shown in (C) for Mϕ to M2 polarisation. (F) GSEA signature showing significantly positive enrichment during Mϕ to M2 polarisation. Supplementary file2 (PDF 1888 KB) [file 18_2024_5261_MOESM2_ESM.pdf]

Figure S3

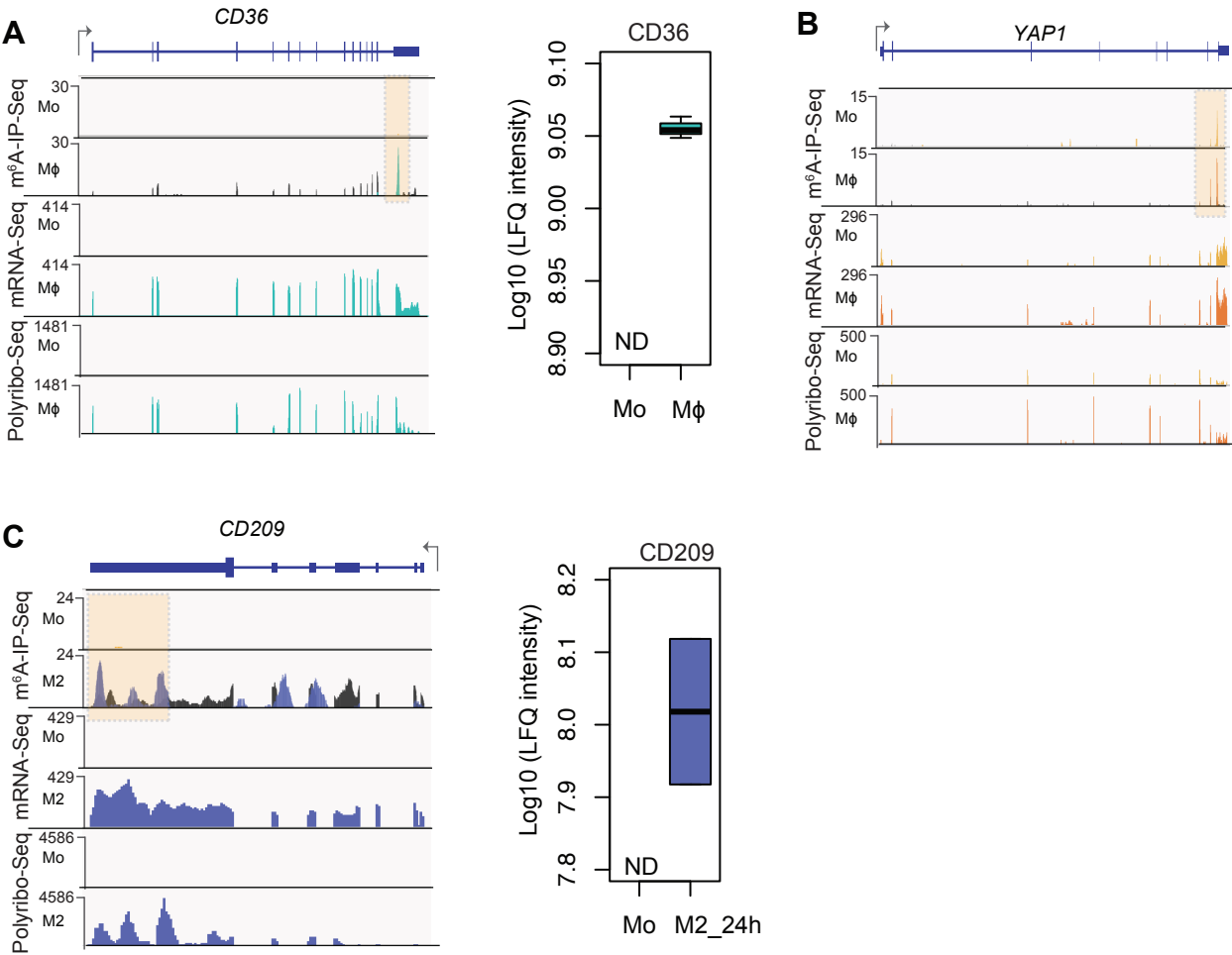

Supplement: Supplementary file 3 — Figure S3. m6A is present in genes that regulate macrophage differentiation and function. (A-C) Left: Coverage plots of m6A-IP-Seq (top), mRNA-Seq (middle) and Polyribo-Seq (bottom) for (A) CD36 in Mϕ, (B) YAP1 in M1 and (C) CD209 and in M2. m6A-IP-Seq tracks show the overlay of input (grey) and IP (yellow, green, orange and blue for Mo, Mϕ, M1 and M2 respectively) data. An m6A peak is highlighted within a dotted box. m6A-IP-Seq coverage plots are displayed in BPM (bins per million reads, Bin size=1). mRNA-Seq and Polyribo-Seq coverage plots are displayed in RPKM (reads per kilobase per million reads). Right: Protein abundance as measured by LC-MS/MS, ND, non-detected. Peptides corresponding to YAP1 were not detected by LC-MS/MS. Supplementary file3 (PDF 531 KB) [file 18_2024_5261_MOESM3_ESM.pdf]

Figure S4

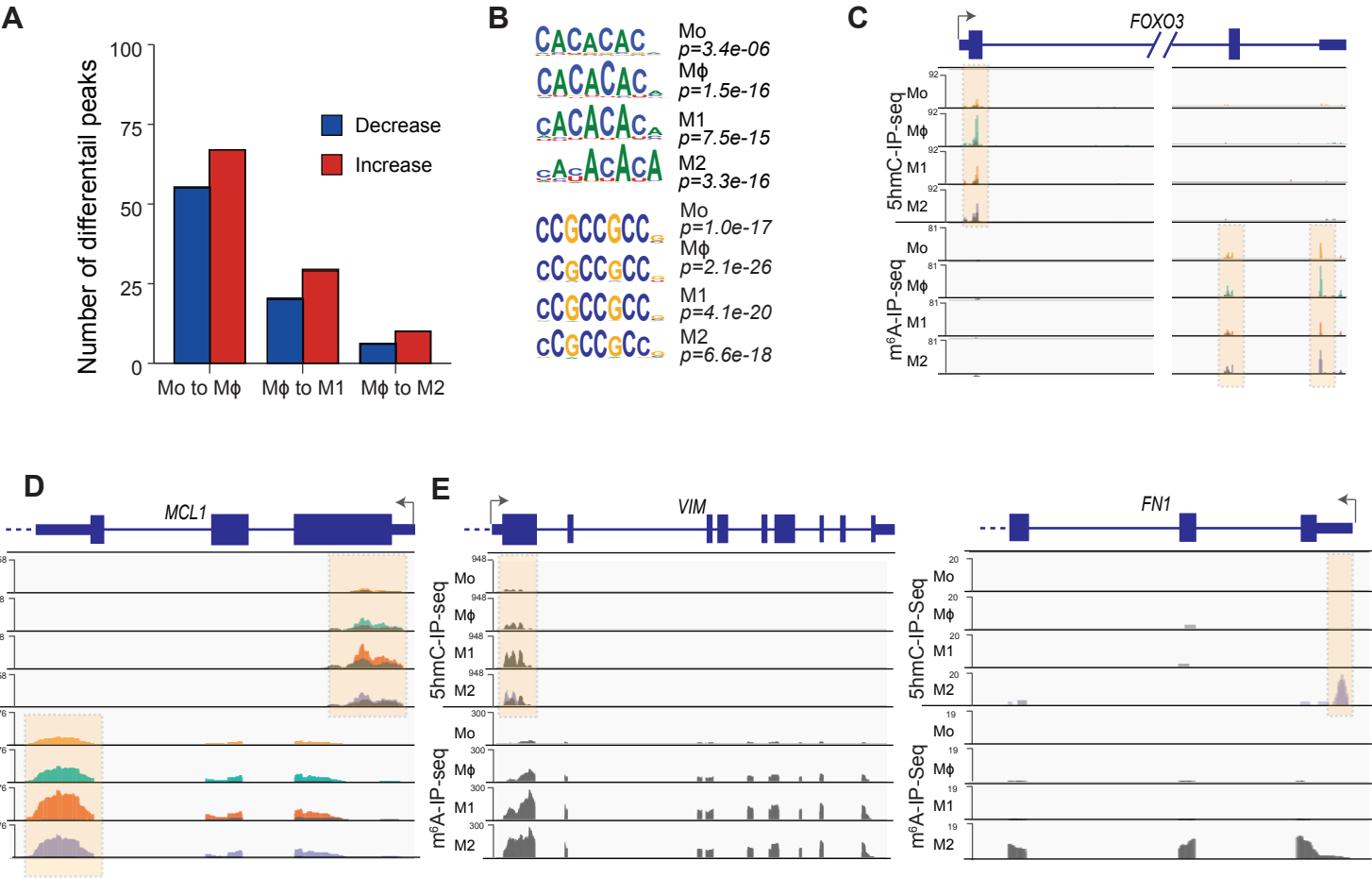

Supplement: Supplementary file 4 — Figure S4. Co-occurence of 5 hmC and m6A in genes that are involved in macrophage differentiation and polarisation. (A) Number of increased (red) and decreased (blue) 5 hmC peaks during macrophage differentiation and polarisation. (B) Significantly enriched sequence motifs identified by 5 hmC-IP-Seq in Mo, Mϕ, M1 and M2. 5 hmC and m6A peaks in FOXO3 (C), MCL1 (D), VIM and FN1 (E) transcripts identified in 5 hmC-IP-Seq (top) and m6A-IP-Seq (bottom) data from Mo, Mϕ, M1 and M2. 5 hmC- and m6A-IP-Seq tracks show the overlay of input and IP data. m6A and 5hmC peaks are highlighted within dotted boxes. 5 hmC- and m6A-IP-Seq coverage plots are displayed in BPM (bins per million reads, Bin size=1). Supplementary file4 (PDF 620 KB) [file 18_2024_5261_MOESM4_ESM.pdf]

Figure S5

A

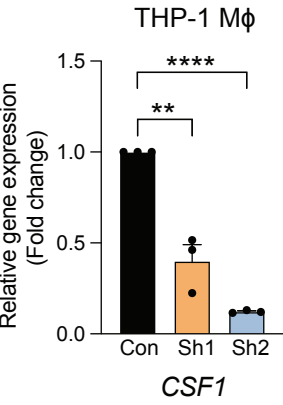

B

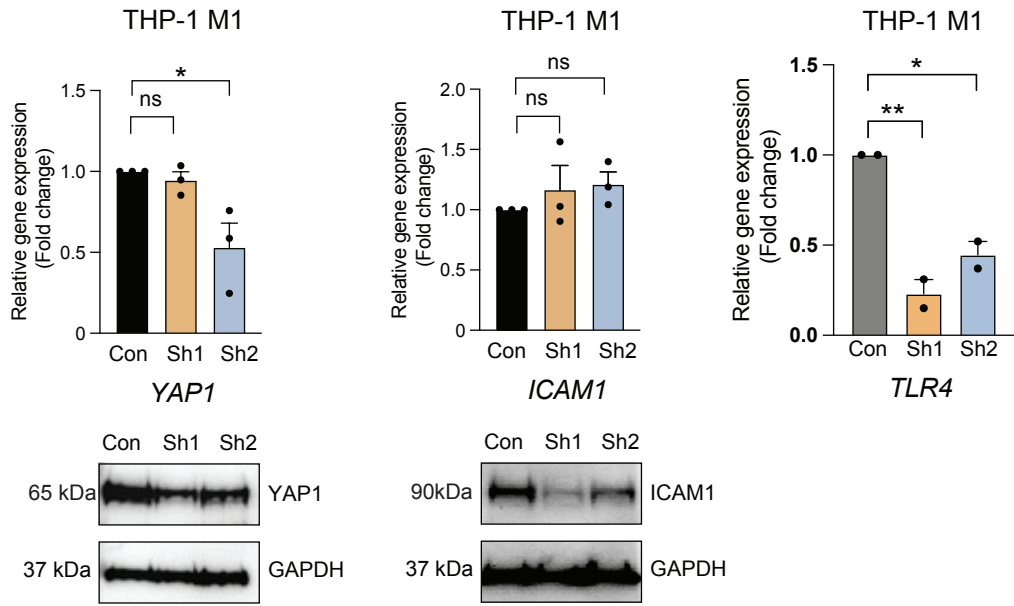

C

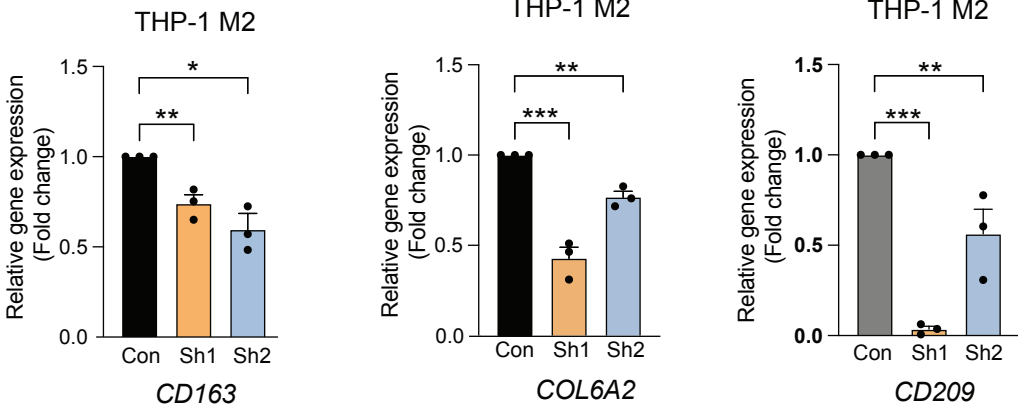

Supplement: Supplementary file 5 — Figure S5. Expression of genes relevant to monocyte and macrophage differentiation and polarisation following METTL3 knockdown. Gene expression changes for CSF1 (A), YAP1, ICAM1, TLR4 (B) and CD163, COL6A2 and CD209 (C) following METTL3 depletion in Mϕ, M1 and M2 cells respectively. (B bottom) western blots showing YAP1 and ICAM1 protein levels following THP-1 transduction with control (Con) or METTL3-specific (Sh1 and Sh2) shRNAs. All data are from at least two independent experiments. Bar plots show mean ± SEM. An unpaired two-tailed Student’s t-test was used to determine significance, denoted by ns, not significant; *, p <0.05; *** and p <0.001. Supplementary file5 (PDF 1062 KB) [file 18_2024_5261_MOESM5_ESM.pdf]

**Figure S6**

**A**

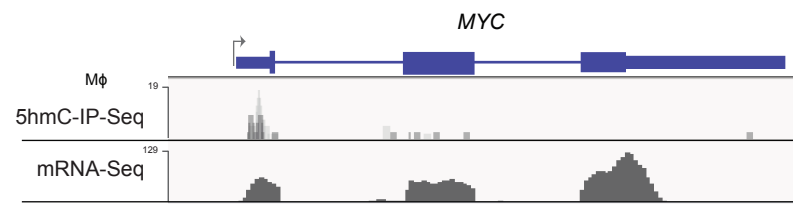

**B**

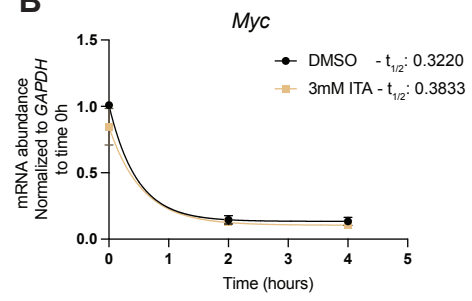

Supplement: Supplementary file 6 — Figure S6. Half-life of MYC mRNA following treatment with Itaconic Acid. (A) Coverage plots of 5 hmC-IP-Seq (top) and mRNA-Seq (bottom) data for MYC in Mϕ. (B) mRNA decay plot for MYC in Itaconic Acid (ITA)-treated Mϕ. Supplementary file6 (PDF 467 KB) [file 18_2024_5261_MOESM6_ESM.pdf]
